# PubChem3D: conformer ensemble accuracy

**DOI:** 10.1186/1758-2946-5-1

**Published:** 2013-01-07

**Authors:** Sunghwan Kim, Evan E Bolton, Stephen H Bryant

**Affiliations:** 1 National Center for Biotechnology Information, National Library of Medicine, National Institutes of Health, Department of Health and Human Services, 8600 Rockville Pike, Bethesda, MD 20894, USA

## Abstract

**Background:**

PubChem is a free and publicly available resource containing substance descriptions and their associated biological activity information. PubChem3D is an extension to PubChem containing computationally-derived three-dimensional (3-D) structures of small molecules. All the tools and services that are a part of PubChem3D rely upon the quality of the 3-D conformer models. Construction of the conformer models currently available in PubChem3D involves a clustering stage to sample the conformational space spanned by the molecule. While this stage allows one to downsize the conformer models to more manageable size, it may result in a loss of the ability to reproduce experimentally determined “bioactive” conformations, for example, found for PDB ligands. This study examines the extent of this accuracy loss and considers its effect on the 3-D similarity analysis of molecules.

**Results:**

The conformer models consisting of up to 100,000 conformers per compound were generated for 47,123 small molecules whose structures were experimentally determined, and the conformers in each conformer model were clustered to reduce the size of the conformer model to a maximum of 500 conformers per molecule. The accuracy of the conformer models before and after clustering was evaluated using five different measures: root-mean-square distance (RMSD), shape-optimized shape-Tanimoto (*ST*^*ST-opt*^) and combo-Tanimoto (*ComboT*^*ST-opt*^), and color-optimized color-Tanimoto (*CT*^*CT-opt*^) and combo-Tanimoto (*ComboT*^*CT-opt*^). On average, the effect of clustering decreased the conformer model accuracy, increasing the conformer ensemble’s RMSD to the bioactive conformer (by 0.18 ± 0.12 Å), and decreasing the *ST*^*ST-opt*^, *ComboT*^*ST-opt*^, *CT*^*CT-opt*^, and *ComboT*^*CT-opt*^ scores (by 0.04 ± 0.03, 0.16 ± 0.09, 0.09 ± 0.05, and 0.15 ± 0.09, respectively).

**Conclusion:**

This study shows the RMSD accuracy performance of the PubChem3D conformer models is operating as designed. In addition, the effect of PubChem3D sampling on 3-D similarity measures shows that there is a linear degradation of average accuracy with respect to molecular size and flexibility. Generally speaking, one can likely expect the worst-case minimum accuracy of 90% or more of the PubChem3D ensembles to be 0.75, 1.09, 0.43, and 1.13, in terms of *ST*^*ST-opt*^, *ComboT*^*ST-opt*^, *CT*^*CT-opt*^, and *ComboT*^*CT-opt*^, respectively. This expected accuracy improves linearly as the molecule becomes smaller or less flexible.

## Background

The advent of combinatorial chemistry and high-throughput screening technology has made it possible to perform a rapid test of biological activity on a vast number of small molecules, generating a massive amount of biological activity data. While this explosion of information presents scientists with great opportunities to facilitate the identification of potential drug candidates and chemical probes, its benefit is enhanced when this data is combined with that of the others and made available to all. Dissemination of such information requires a public repository that collects and stores the heterogeneous data from various contributors. An example of such a repository is PubChem [1-4] (http://pubchem.ncbi.nlm.nih.gov), launched in 2004 as a component of the Molecular Libraries Roadmap Initiatives of the U.S. National Institutes of Health. PubChem archives biological activity screening data and other information from diverse data sources and offers its contents free of charge to the biomedical research community, facilitating research that benefits human health.

PubChem consists of three primary databases: Substance, Compound, and BioAssay. The PubChem Substance database contains sample descriptions provided by individual depositors and the PubChem Compound database contains the unique standardized chemical structure contents extracted from the PubChem Substance database. The PubChem BioAssay database contains biological assay descriptions and results. As of June 2012, PubChem contains more than 92 million substance descriptions, 32 million unique small molecules, 600 thousand biological assays, and 170 million biological assay outcomes (each outcome is a set of results from a substance being tested in an assay). PubChem provides search, analysis, and download tools for the efficient use of this vast amount of chemical information. Many of these tools exploit the concept of molecular similarity at some level. One method in which PubChem evaluates chemical similarity between two molecules is to use a two-dimensional (2-D) dictionary-based fingerprint [5] and the Tanimoto equation [6,7]:

(1) $$\mathrm{Tanimoto}=\frac{\mathrm{AB}}{A+B-\mathrm{AB}}$$

where A and B are the respective counts of the set binary fingerprint bits for the two molecules and AB is the count of set bits in common to both molecules. Because the 2-D molecular similarity computation is very fast (typically at a rate of one million pair-wise comparisons per second per CPU core), it is appropriate for searching a large database like PubChem. However, there are many diverse chemical structures with similar biological efficacies against targets available in PubChem that can be difficult to interrelate using traditional 2-D similarity methods [8-11]. To assist in biological activity analysis of these molecules, a new layer called PubChem3D [8-15] was added to PubChem.

PubChem3D generates a 3-D conformer model description for each record in the PubChem Compound database, when it satisfies the following conditions [13]: (1) not too large (with 50 or fewer non-hydrogen atoms); (2) not too flexible (with no more than 15 rotatable bonds); (3) has only a single covalent unit (*i.e.*, not a salt or mixture); (4) consists of only supported elements (H, C, N, O, F, Si, P, S, Cl, Br, and I); (5) contains only atom-types recognized by the Merck Molecular Force Field (MMFF94s) [16,17]; and (6) five or fewer undefined atom (R,S) and bond (E,Z) stereo centers. This 3-D description can be employed to enhance existing PubChem search and analysis methodologies by means of 3-D similarity [10], helping the user identify useful structure-activity relationships that might go unrecognized by the PubChem 2-D similarity method. A diverse conformer ordering [10] gives a maximal description of the conformational space of a molecule when only a subset of available conformers is used. A pre-computed search per compound record gives immediate access to a set of 3-D similar compounds (called “Similar Conformers” [8]) in PubChem and their respective superpositions, augmenting the complementary “Similar Compounds” relationship, computed using the PubChem 2-D similarity method. Systematic augmentation of PubChem resources to include a 3-D layer provides users with new capabilities to search, subset, visualize, analyze, and download data [10].

All the tools and services in PubChem3D rely upon the quality and applicability of the computationally-derived 3-D conformer models of small molecules. Considering the size of PubChem, all these conformer models by necessity must be pre-computed and stored to allow the user “real-time” access to identify structurally similar conformers and to analyze biological activity patterns. Among many different conformer generation programs that exist [18-24], PubChem3D uses the OMEGA C++ toolkit [25-28] to generate conformer ensembles. In our previous study [13], an optimal set of adjustable parameters were determined to maximize the “accuracy” of OMEGA (*i.e.*, the ability to reproduce experimentally-determined “bioactive” conformations, for example, found in protein-ligand complexes). Using experimentally determined structures of 25,972 small-molecule ligands found in the Protein Databank (PDB) [29], the effects of parameter values used in OMEGA upon the root-mean-square distance (RMSD) between the computationally-derived conformer models and their experimentally-determined bioactive conformations were analyzed in terms of the non-hydrogen (heavy) atom count (*N*_*NHA*_) and effective rotor count (*N*_*ER*_), as measures of molecular size and flexibility, respectively [30]. Note that *N*_*ER*_ is given by the following equation and takes into account molecular flexibility due to rotatable bonds and ring flexibility:

(2) $$N_{\text{ER}}=N_{R}+\frac{N_{\text{NARA}}}{5}$$

where *N*_*R*_ is the number of rotatable bonds, and *N*_*NARA*_ is the number of non-aromatic *sp*^3^-hybridized ring atoms. The root-mean-square distance (RMSD) accuracy of the computationally-derived conformer models was found to strongly depend on molecular size and flexibility, leading to the following formula [13] that estimates the worst-case RMSD accuracy of nearly all the conformer models using only the values of *N*_*NHA*_ and *N*_*ER*_:

(3) $$\mathrm{RMS}D^{\mathrm{pred}}=0.219+0.0099\times N_{\mathrm{NHA}}+0.040\times N_{\mathrm{ER}}$$

where *RMSD*^*pred*^ is the predicted upper limit of the RMSD accuracy to ensure at least 90% of conformer models generated by OMEGA using the selected PubChem parameter set for the 25,972 PDB ligands had at least one “bioactive” conformer whose RMSD distance from the experimentally determined conformation was closer than the value predicted using Equation (3).

Using this accuracy-calibrated OMEGA parameter set, PubChem3D generates up to 100,000 conformers for each chemical structure stereo configuration. However, it is still not feasible to store all the conformers in a database and use them in a very efficient way. Therefore, the conformers in each ensemble are sampled through clustering with the *RMSD*^*pred*^ value, after rounding to the nearest 0.2 increment as defined in the following equation,

(4) $$\mathrm{RMS}D^{\mathrm{cluster}}=\frac{\text{int}\;\left(0.5+\mathrm{RMS}D^{\mathrm{pred}}*5\right)}{5}$$

where “int( )” gives the whole number, irrespective of any remaining fraction and where this RMSD threshold is referred to as *RMSD*^*cluster*^ to emphasize its usage for clustering purposes (rather than accuracy prediction). Each sampled conformer represents a cluster containing all conformers within the designated RMSD threshold, thus reducing the count of conformers per conformer model. If the conformer model after cluster sampling has more than a maximum of 500 conformers, it is re-clustered using an *RMSD*^*cluster*^ value incremented by a further 0.2. This process is repeated as many times as necessary to reduce the overall conformer count to 500 or less. Although this clustering process makes the conformer models more manageable in size and better suited for a large database such as PubChem, it may be accompanied with an undesirable loss of overall accuracy of the conformer model. Therefore, in the present study, we investigated the effect of the conformer model clustering upon the accuracy of the conformer models as a follow-up to our previous study [13] in order to address key questions as to the performance of PubChem 3D sampled conformers to reproduce “bioactive” ligand geometries: as a function of molecular size and flexibility, with respect to the established PubChem3D similarity measures, and with an eye towards their expected performance relative to biological activity data analysis.

## Results and discussion

### Molecular size and flexibility of the MMDB ligands

This study considers 47,123 small molecules with experimental 3-D coordinates available from the Molecular Modeling Database (MMDB) [31] deposition in PubChem (Additional file 1). The molecular connectivity of these MMDB ligands is derived from the 3-D coordinates of the protein-bound small molecules taken from PDB [29] records. Note that the “experimental” structures of small molecules in PDB are known to, at times, have non-trivial issues or uncertainty concerning their precise chemical identity, protein binding geometry, or crystal structure location [13,32-36]. The present study largely ignores such potential issues and considers all the 3-D ligand structures as experimental facts.

**Figure 1:**
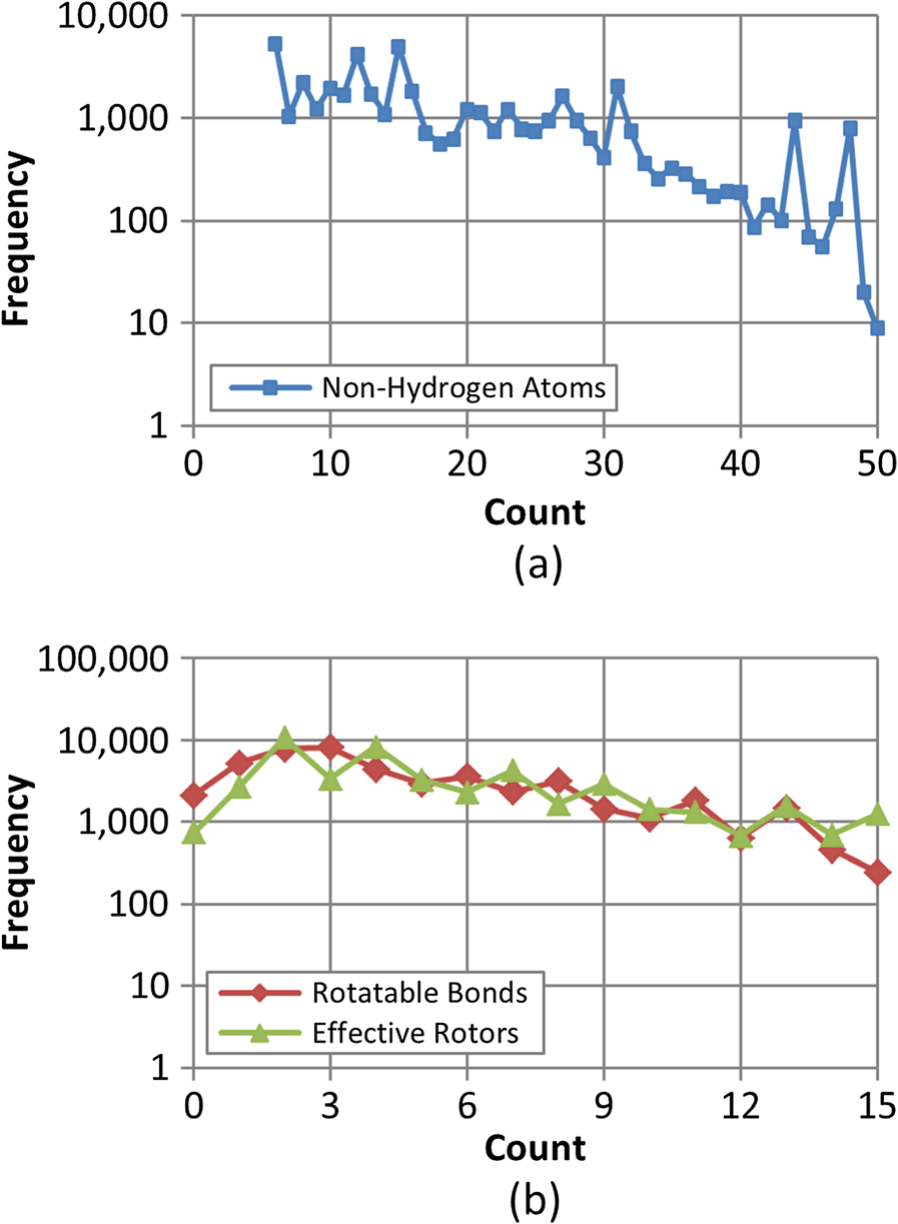
**Molecular size and flexibility of bioactive ligand data set.** Frequency of **(a)** the non-hydrogen (heavy) atom count and **(b)** the rotatable bond count and the effective rotor count for the 47,123 experimentally determined “bioactive” ligand structures in the MMDB data set. The effective rotor counts were binned to the nearest whole numbers.

The effects of conformer ensemble clustering upon the accuracy of the conformer ensemble were analyzed as a function of *N*_*NHA*_ and *N*_*R*_ (as measures of molecular size and flexibility, respectively). Additionally, *N*_*ER*_ [Equation (2)] was also employed to represent molecular flexibility. Although the value of *N*_*ER*_ is not necessarily an integer, it was rounded to the nearest integer in the present study. Figure 1 shows the distributions of the values of *N*_*NHA*_, *N*_*R*_, and *N*_*ER*_ for the experimental structures for the 47,123 MMDB ligands considered. On average, the structures had 18.3 ± 10.5 non-hydrogen (heavy) atoms, 4.7 ± 3.5 rotatable bonds, and 5.4 ± 3.7 effective rotors. Approximately 90% of the ligands had less than 31 non-hydrogen atoms, 9 rotatable bonds, and 10 effective rotors.

**Figure 2:**
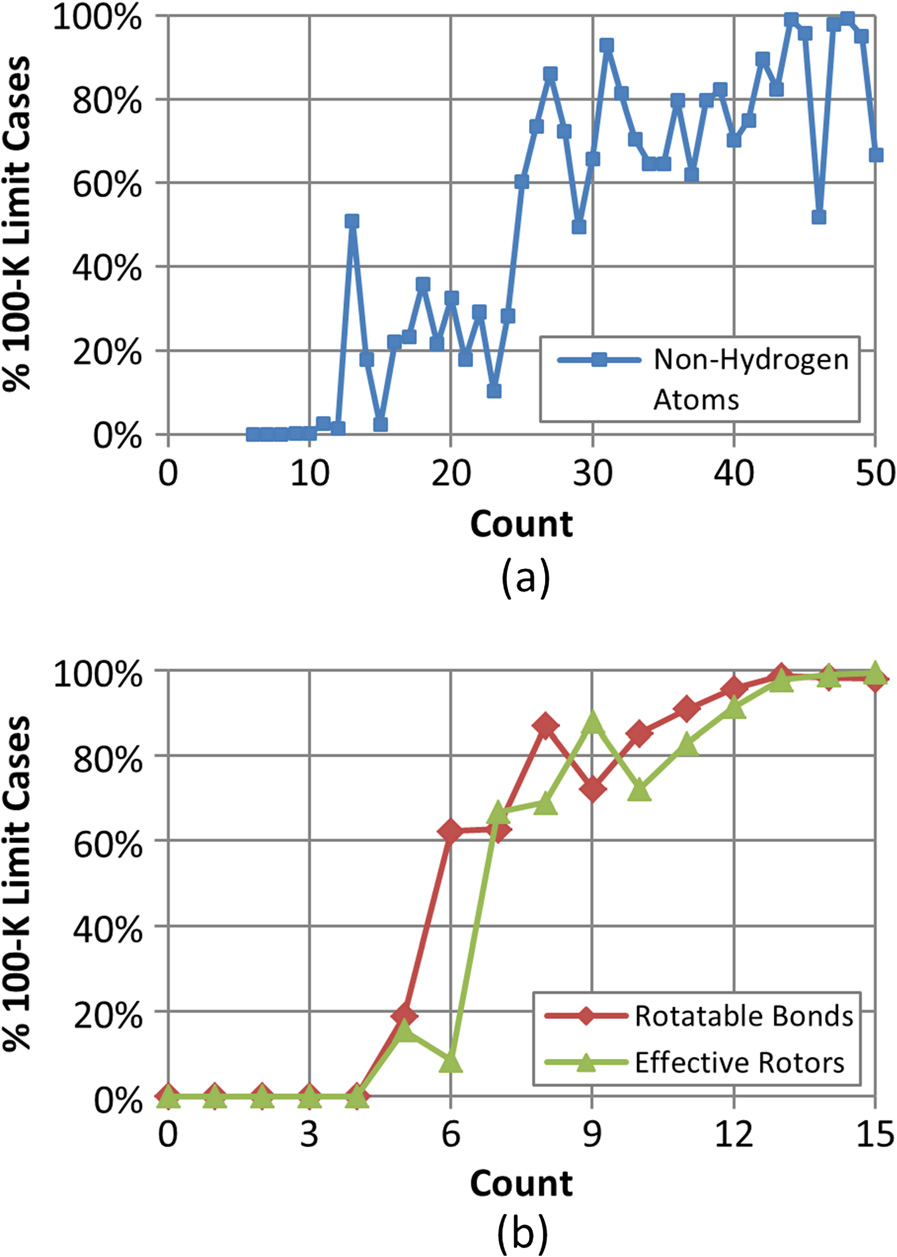
**The 100-K limit cases *vs*. molecular size and flexibility.** The fraction of the 47,123 MMDB ligands reaching the limit of 100,000 conformers per compound during the conformer generation step as a function of: **(a)** the non-hydrogen atom count and **(b)** the rotatable bond count and the effective rotor count.

PubChem3D generates a maximum of 100,000 conformers per compound stereo configuration for efficiency considerations. Reaching this “100-K” limit suggests a loss in conformational space considered, possibly resulting in less accurate conformer models [13]. As shown in Figure 2, the fraction of the molecules hitting the 100-K limit increases rapidly as the molecule becomes larger and more flexible. This suggests that, beyond 25 heavy atoms and six rotatable bonds, exploration of conformational space in some PubChem3D conformer models may be truncated due to this limitation.

### RMSD clustering threshold

**Figure 3:**
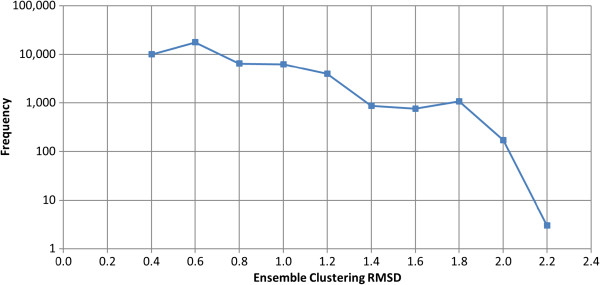
***RMSD***^***cluster***^**conformer model sampling threshold.** The frequency of the RMSD clustering threshold (*RMSD*^*cluster*^) values used during the conformer clustering procedure for the 47,123 MMDB ligand conformer models.

After generating conformers for each molecule, PubChem3D samples the conformers in each ensemble, using an RMSD threshold determined according to Equation (3) and Equation (4). Figure 3 shows the distribution of the *RMSD*^*cluster*^ values used to cluster the conformers in the conformer ensemble for the 47,123 MMDB ligands considered in the present study. The *RMSD*^*cluster*^ values range from 0.4 Å to 2.2 Å (in discrete 0.2 Å increments), with an average and standard deviation of 0.75 Å ± 0.33 Å. Approximately 85% of the 47,123 PDB ligands have an *RMSD*^*cluster*^ value of ≤ 1.0 Å.

**Figure 4:**
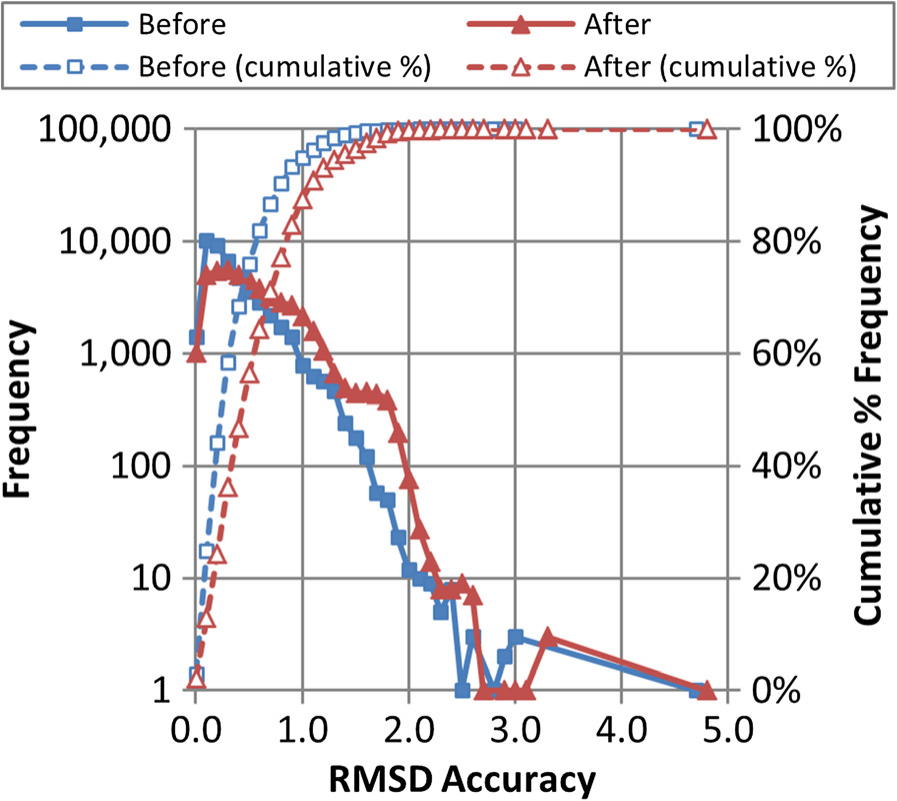
**Overall RMSD accuracy of the conformer models.** The RMSD accuracy (binned in 0.1 Å increments) of the 47,123 MMDB ligand conformer models to the corresponding experimental 3-D structure, before and after the conformer model clustering procedure, by frequency and cumulative % frequency.

The distributions of the RMSD “accuracy” of the resul-ting conformer ensembles to the experimental PDB ligand geometries are shown in Figure 4 and their average and standard deviations are summarized in Table 1. It is important to note that, for the purpose of this study, the RMSD “accuracy” value of a computationally-derived conformer ensemble to the experimental “bioactive” conformation is defined as the single best (*i.e.*, least) non-hydrogen atom-pairwise RMSD between the experimentally determined 3-D conformation PDB ligand and a 3-D conformer in the ensemble. This RMSD “accuracy” should not be confused with the clustering RMSD (*RMSD*^*cluster*^).

**Table 1:** Summary statistics of overall conformer model accuracy

**Accuracy measure**	**Before clustering**		**After clustering**		**Difference**	
	***μ***	***σ***	***μ***	***σ***	***μ***	***σ***
RMSD (Å)	0.39	0.24	0.57	0.36	0.18	0.12
*ST*^*ST-opt*^	0.95	0.06	0.91	0.09	−0.04	0.03
*ComboT*^*ST-opt*^	1.74	0.20	1.57	0.27	−0.16	0.09
*CT*^*CT-opt*^	0.85	0.13	0.75	0.17	−0.09	0.05
*ComboT*^*CT-opt*^	1.77	0.20	1.61	0.27	−0.15	0.09

Overall average (*μ*) and standard deviation (*σ*) of the accuracy of the 47,123 MMDB ligand conformer models to the corresponding experimental 3-D structure before and after conformer sampling clustering.

As expected, the PubChem3D conformer sampling procedure results in a loss of the conformer ensemble RMSD accuracy relative to experiment. On average, this overall loss is 0.18 Å (from 0.39 Å to 0.57 Å). The standard deviation of this average also increases by 0.12 Å (from 0.24 Å to 0.36 Å) and may reflect the rounding of *RMSD*^*cluster*^ to the nearest 0.2 increment, potentially suggesting the ± 0.1 nature of such a change. In the aggregate, 90% of all conformer models in this study reflect RMSD accuracies better than 1.1 Å after clustering.

In the study by Hawkins *et al.*[18], an RMSD value of 1.25 Å or less was employed as the definition of a “close” reproduction of the experimental conformation. They also pointed out that an RMSD of 2.0 Å could have been used as a cut-off because it is a common upper bound for successful reproduction of an experimental structure in molecular docking. With these criteria in mind, the after-clustering conformer models in the present study may be considered to be of high quality, although the choice of the cut-off for “close” reproduction of the experimental structure is still arbitrary and subjective.

**Figure 5:**
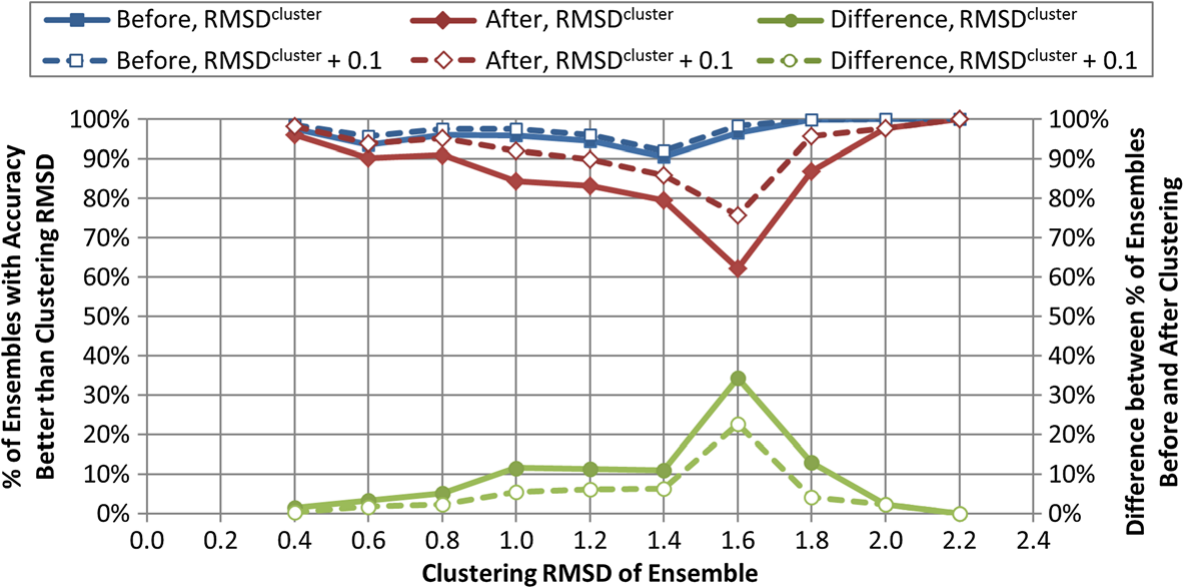
**Conformer models with accuracy better than *RMSD***^***cluster***^**.** The fraction of the conformer models of the 47,123 MMDB ligands with an RMSD to the corresponding experimental 3-D structure less than the RMSD clustering threshold (*RMSD*^*cluster*^) (solid line) and *RMSD*^*cluster*^ + 0.1 Å (dashed line).

Figure 5 illustrates the percentage of the conformer models with accuracy better than the *RMSD*^*cluster*^ value (*i.e.*, with an RMSD accuracy value less than *RMSD*^*cluster*^) before and after the conformer sampling procedure. For comparison purposes, those conformer models with the accuracy better than *RMSD*^*cluster*^ + 0.1 Å are also included to show the effects of the rounding of *RMSD*^*pred*^ to the nearest 0.2 (*i.e.*, *RMSD*^*cluster*^). It is important for two reasons to note that more than 90% of the conformer models *before clustering* have an RMSD accuracy better than *RMSD*^*cluster*^. Firstly, this shows that, although the majority of conformer models with more than 25 heavy atoms and six rotatable bonds hit the 100-K limit in conformer generation as shown in Figure 2, there is no significant adverse effect on the conformer model accuracy beyond that already reflected by Equation (4). Secondly, this indicates the resilience of OMEGA to generate biologically relevant conformers, favoring a breadth-first exploration of conformer space as a function of energy threshold (*i.e.*, considering low-energy conformational spaces first), thus ensuring an even coverage of conformer space up to the PubChem3D 100-K conformer limit in the conformer generation phase. For the remaining cases where the conformer models before clustering are not more accurate than the *RMSD*^*cluster*^ threshold, OMEGA does not even come close to reproducing the experimental geometry using the PubChem3D choice of parameters, considering that an increase of 0.1 Å from *RMSD*^*cluster*^ does not find many additional “missed” pre-clustered conformer mo-dels, as Figure 5 shows. Whereas potential reasons for this are numerous, one can likely attribute it to: improper perception of atom hybridization or charge state from PDB atom coordinates in the MMDB deposition (leading to an inaccurate chemical structure, as PDB records tend not to include hydrogen atom or bond order information); some combination of errors, uncertainty, or omissions in the PDB ligand information (as mentioned previously); or a general inability of OMEGA to reproduce some “bioactive” 3-D chemical structure configurations.

As shown in Figure 5, *after clustering*, the fraction of the conformer models with accuracy better than *RMSD*^*cluster*^ decreased by no more than 12% in general, except for *RMSD*^*cluster*^ = 1.6 Å (34%). When the conformer model accuracy was predicted in a more conservative way using the limit *RMSD*^*cluster*^ + 0.1 Å (rather than *RMSD*^*cluster*^), the difference between the conformer models with accuracy better than this limit *before* and *after* clustering was no more than 6%, except for *RMSD*^*cluster*^ = 1.6 Å (23%), sho-wing that the realized sampling effects are local in nature. It appears that most of the structures with decreased conformer model accuracy at *RMSD*^*cluster*^ = 1.6 Å are simply due to an unfortunate culmination of pronounced partition-based clustering edge-effects for a set of flexible di- and tri-phosphate containing structures. In other words, for these particular computationally-derived conformer models, a conformation most similar to the experimental structures happened to be near the boundaries of the clusters generated with the *RMSD*^*cluster*^ = 1.6 Å, and therefore, they were not included in the conformer models after the clustering procedure. As a result, clustering a given conformer model using a different conformer ordering with the same clustering procedure could have yielded results closer to the pre-clustering result.

**Figure 6:**
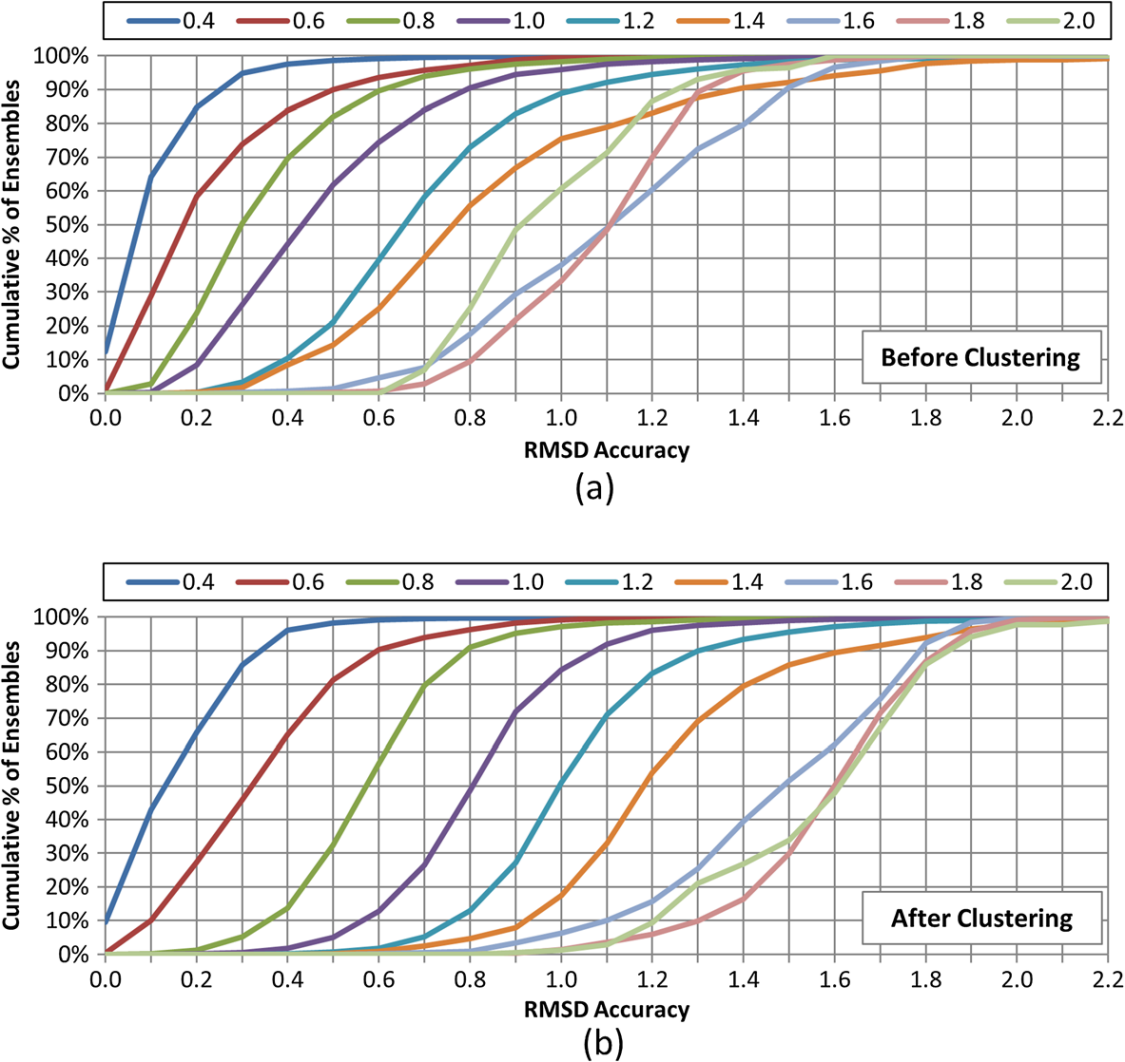
**Accuracy of conformer models as a function of *RMSD***^***cluster***^**.** The cumulative % distribution of the RMSD accuracy (binned in 0.1 Å increments) of the 47,123 MMDB ligand conformer models to the corresponding experimental 3-D structure as a function of RMSD clustering threshold (*RMSD*^*cluster*^): **(a)** before clustering and **(b)** after clustering. [Note the three conformer models at 2.2 Å were removed from this and some other figures for clarity].

Figure 6 illustrates the cumulative % distribution of the RMSD accuracy of the conformer models for each discrete value of *RMSD*^*cluster*^. As mentioned above, the *RMSD*^*cluster*^ value determination using Equation (3) was intended to ensure that 90% of conformer models have an RMSD accuracy below *RMSD*^*cluster*^*before* sampling; however, the RMSD accuracy *after* sampling to ensure 90% of conformers are found is expected to be within the range of *RMSD*^*cluster*^ ± 0.1, when considering the effects of the rounding of *RMSD*^*cluster*^ to the nearest increment of 0.2 [as performed in Equation (4)]. If one looks across the 90% line in panel (a) of Figure 6, the RMSD accuracies of 90% of the conformer models *before clustering* are smaller than *RMSD*^*cluster*^ for the entire range in general, with almost no difference at *RMSD*^*cluster*^ = 1.4 Å. The 90% levels of the *after-clustering* RMSD accuracies [in panel (b) of Figure 6], are within the expected range in general, except for *RMSD*^*cluster*^ = 1.4 Å and 1.6 Å, where the RMSD accuracy for 90% of the conformer models is not reached until 1.6 Å and 1.8 Å, respectively.

One readily notices for each *RMSD*^*cluster*^ value in Figure 6 that conformer model clustering shifts the cumulative % distribution curves toward the right-hand side, indicating a decrease in the conformer model accuracy as a result of the PubChem sampling procedure. Looking at the 90% level of conformer models *before* and *after* clustering, there are some variances in the change of the conformer model accuracy, depending on the *RMSD*^*cluster*^ value. For example, the difference between the RMSD accuracies at the 90% level *before* and *after* clustering with *RMSD*^*cluster*^ of 0.4 Å and 0.6 Å is 0.1 Å (0.25 Å *vs.* 0.35 Å for *RMSD*^*cluster*^ = 0.4 Å, and 0.5 Å *vs.* 0.6 Å for *RMSD*^*cluster*^ = 0.6 Å). However, for the *RMSD*^*cluster*^ values between 0.6 Å and 1.6 Å, the corresponding differences range between 0.2 Å and 0.3 Å. In general, it is very reassuring to see that most of these conformer models at the 90% level are within the expected range for most *RMSD*^*cluster*^ values. Although sampling by its very nature will increase the distance between conformers, this increase does not appear to severely impact the accuracy of the conformer models in PubChem.

### Comparison of ensemble accuracy measures

Evaluation of the conformer model accuracy using RMSD is an intuitive and convenient choice, as the conformer model clustering in PubChem3D uses an RMSD value as a clustering threshold; however, in practice, PubChem3D primarily uses three measures in 3-D similarity comparison between molecules: shape-Tanimoto (ST), color-Tanimoto (CT), and combo-Tanimoto (ComboT) [37-40]. Therefore, the present study also employed PubChem3D similarity measures as additional conformer model accuracy measures. The ST [37-40] similarity measure, which quantifies the shape similarity between molecules, is defined as the following equation:

(5) $$\mathrm{ST}=\frac{V_{\mathrm{AB}}}{V_{\mathrm{AA}}+V_{\mathrm{BB}}-V_{\mathrm{AB}}}$$

where *V*_*AA*_ and *V*_*BB*_ are respective self-overlap volume of the two molecules, and *V*_*AB*_ is the overlap volume between the two molecules. The CT [37,38] similarity measure, on the other hand, evaluates the pharmacophore feature similarity between molecules, by comparing the 3-D orientation of fictitious atoms (also called feature atoms) representing six functional group types (hydrogen-bond donors, hydrogen-bond acceptors, cations, anions, hydrophobes, and rings) by means of the equation:

(6) $$\mathrm{CT}=\frac{\sum_{f}V_{\mathrm{AB}}^{f}}{\sum_{f}V_{\mathrm{AA}}^{f}+\sum_{f}V_{\mathrm{BB}}^{f}-\sum_{f}V_{\mathrm{AB}}^{f}}$$

where the index “*f*” is one of the six functional-group types, *V*_*AA*_^*f*^ and *V*_*BB*_^*f*^ are the self-overlap volume for the functional group type “*f*” of the two molecules, respectively, and *V*_*AB*_^*f*^ is the overlap volume for the functional group type “*f*” between the molecules. The ComboT [37,38] similarity measure, which is defined as the arithmetic sum of the ST and CT scores, allows one to consider the two different similarities simultaneously. Because both the ST and CT scores range from 0 (for no similarity) to 1 (for identical molecules), the ComboT score ranges from 0 to 2 (without normalization, due to pre-existing convention).

The present study used two different approaches to compute these three 3-D similarity scores: the shape-optimized (or ST-optimized) approach and feature-optimized (or CT-optimized) approach. In the shape-optimized approach, the superposition of two molecules is optimized to have a maximum ST score and then the CT score is computed in that shape-optimized alignment. In the feature-optimized approach, the color and shape of the two conformers will be considered simultaneously to find the best superposition between them, as in the current version of ROCS [37]. In the present paper, the shape-optimized and feature-optimized methods are denoted using the superscripts “ST-opt” and “CT-opt”, respectively. As a result, there are six different 3-D similarity scores (*i.e.*, *ST*^*ST-opt*^, *CT*^*ST-opt*^, *ComboT*^*ST-opt*^, *ST*^*CT-opt*^, *CT*^*CT-opt*^, and *ComboT*^*CT-opt*^). Along with RMSD, four of these six scores are used to analyze the accuracy of the clustered conformer models relative to the experimentally determined 3-D geometries: *ST*^*ST-opt*^, *ComboT*^*ST-opt*^, *CT*^*CT-opt*^, and *ComboT*^*CT-opt*^.

**Figure 7:**
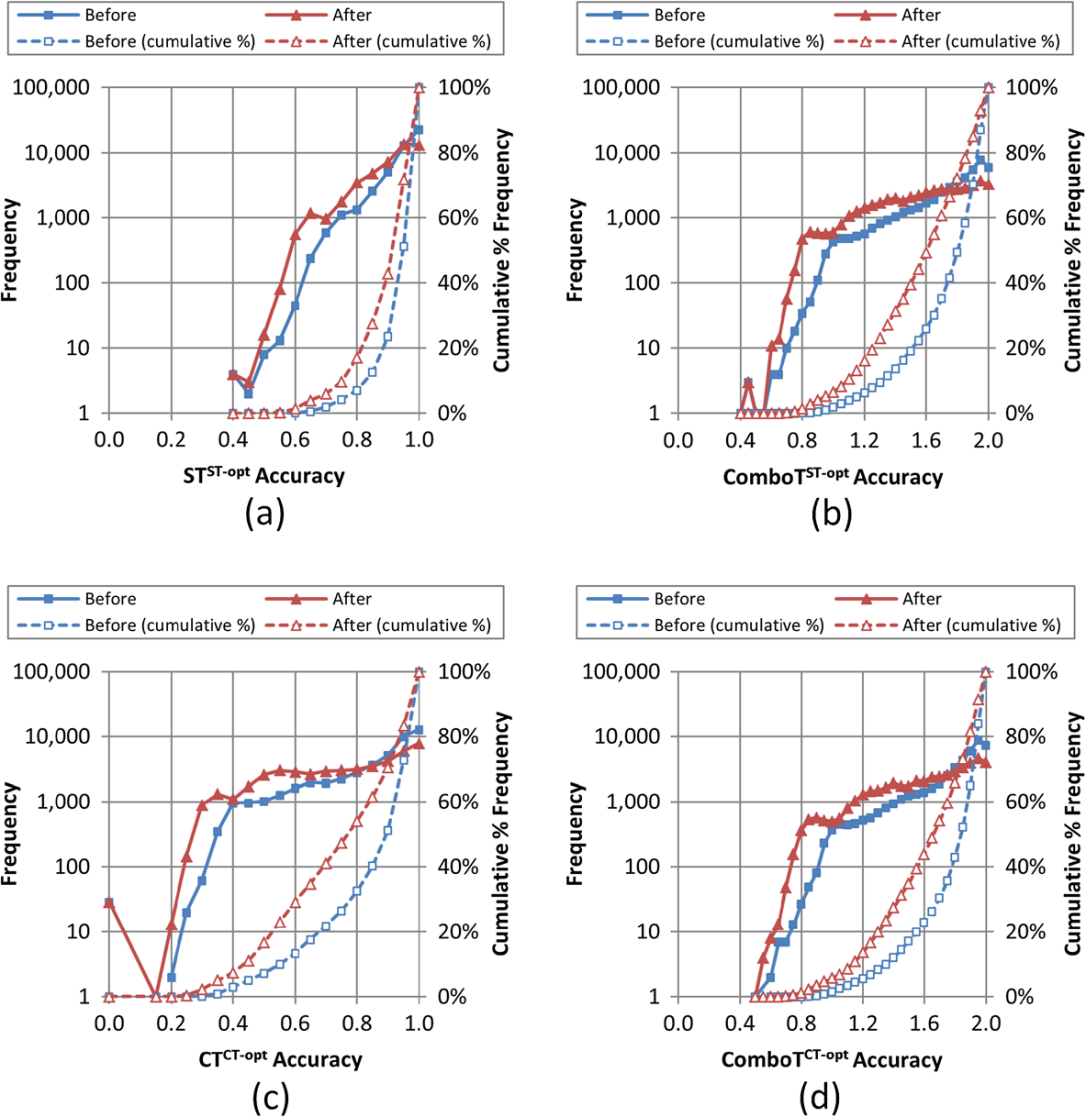
**Overall 3-D similarity accuracy of the conformer models.** The accuracy (binned in 0.05 increments) of the 47,123 MMDB ligand conformer models to the corresponding experimental 3-D structure, before and after the conformer model clustering procedure, by frequency and cumulative % frequency for the 3-D similarity metrics: **(a)***ST*^*ST-opt*^, **(b)***ComboT*^*ST-opt*^, **(c)***CT*^*CT-opt*^, and **(d)***ComboT*^*CT-opt*^.

As shown so far in this study, the conformer sampling procedure decreases ensemble accuracy to reproduce experimentally determined ligand geometries, resulting in an increase in the RMSD values. This loss in accuracy is also seen for the four 3-D similarity values as shown in Table 1. On average, whereas the clustering increases the RMSD value of the conformer ensemble by 0.18 ± 0.12 Å, it decreases the *ST*^*ST-opt*^, *ComboT*^*ST-opt*^, *CT*^*CT-opt*^, and *ComboT*^*CT-opt*^ scores by 0.04 ± 0.03, 0.16 ± 0.09, 0.09 ± 0.05, and 0.15 ± 0.09, respectively. Although the *ComboT*^*CT-opt*^ values are (in the aggregate) slightly greater than the *ComboT*^*ST-opt*^ values, the decreases of the two similarity measures upon clustering are nearly identical in magnitude to each other, suggesting a general insensitiveness of the *ComboT* scores to the optimization type. Perhaps more interesting is the relatively small change in the *ST*^*ST-opt*^ average of 0.04, whereas the *CT*^*CT-opt*^ average difference is more than twice as large, indicating a much greater sensitivity of *CT*^*CT-opt*^ to clustering. This is not surprising, as shape is less discriminating than features (*e.g.*, with a nitrogen atom and carbon atom being nearly identical from a shape perspective but completely different in their capability to make intermolecular interactions). As shown in Figure 7, *after* the conformer sampling procedure, 90% of all the conformer models had accuracies better than 0.75, 1.09, 0.43, and 1.13, in terms of *ST*^*ST-opt*^, *ComboT*^*ST-opt*^, *CT*^*CT-opt*^, and *ComboT*^*CT-opt*^, respectively*.*

**Figure 8:**
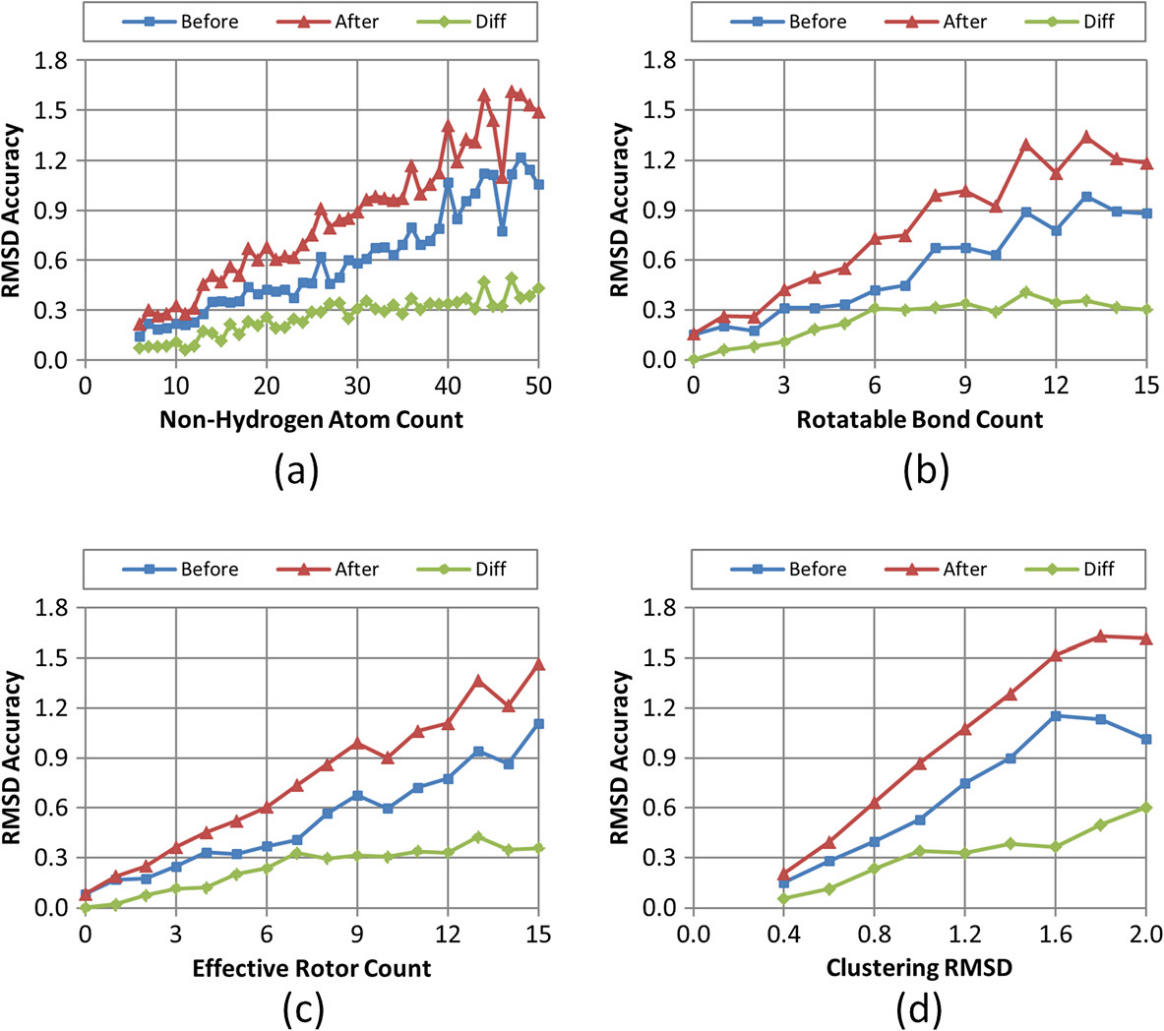
**Average RMSD accuracy as a function of the molecular size, flexibility, and *RMSD***^***cluster***^**.** The average conformer model RMSD accuracy of the 47,123 MMDB ligand conformer models to the corresponding experimental 3-D structures, before and after the conformer model clustering procedure, as a function of: **(a)** the non-hydrogen atom count, **(b)** the rotatable bond count, **(c)** the effective rotor count, and **(d)** the RMSD clustering threshold (*RMSD*^*cluster*^).

**Figure 9:**
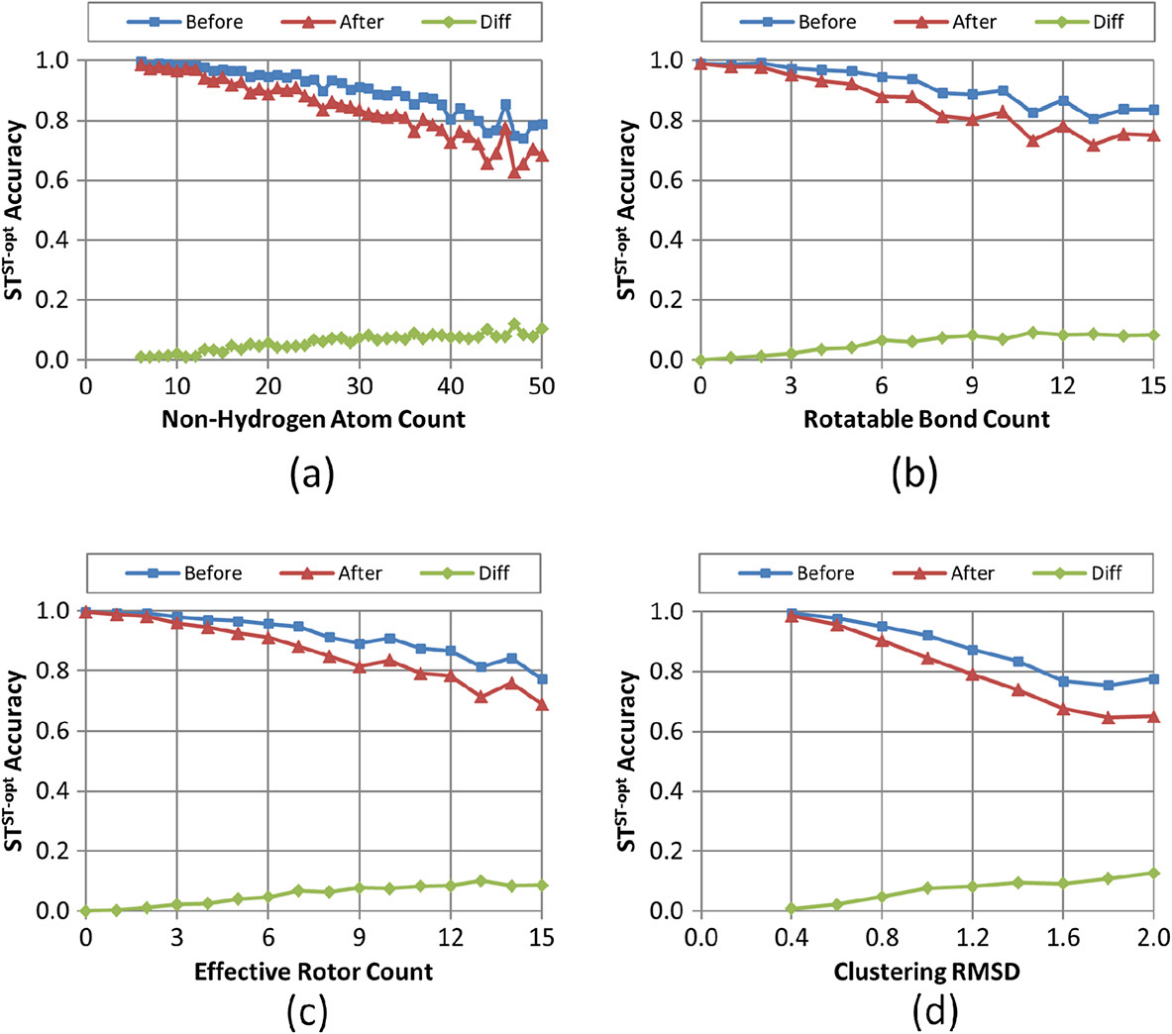
**Average *ST***^***ST-opt***^**accuracy as a function of the molecular size, flexibility, and *RMSD***^***cluster***^**.** The average conformer model shape-optimized shape-Tanimoto (*ST*^*ST-opt*^) accuracy of the 47,123 MMDB ligand conformer models to the corresponding experimental 3-D structure, before and after the conformer model clustering procedure, as a function of: **(a)** the non-hydrogen atom count, **(b)** the rotatable bond count, **(c)** the effective rotor count, and **(d)** the RMSD clustering threshold (*RMSD*^*cluster*^).

**Figure 10:**
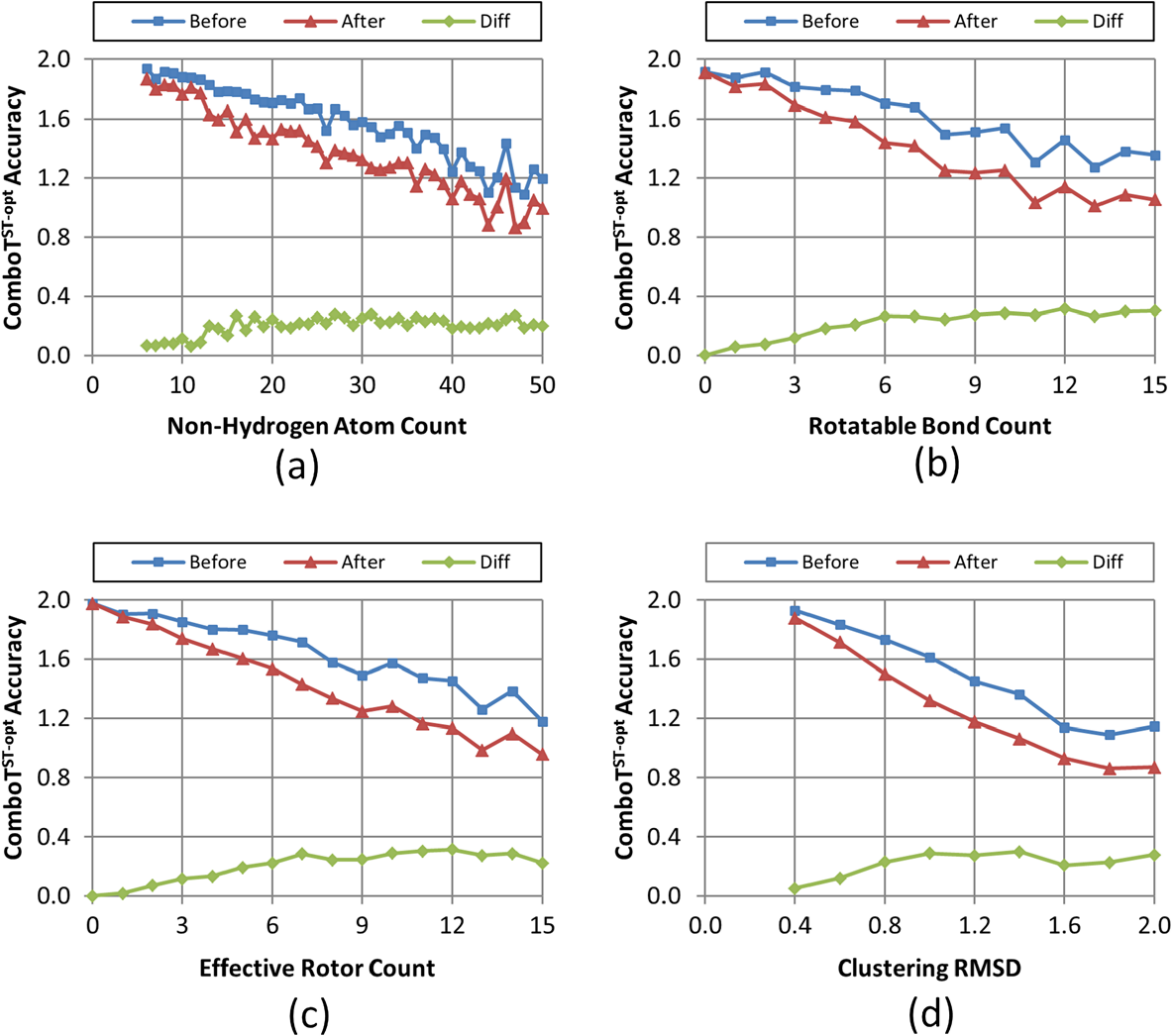
**Average *ComboT***^***ST-opt***^**accuracy as a function of the molecular size, flexibility, and *RMSD***^***cluster***^**.** The average conformer model shape-optimized combo-Tanimoto (*ComboT*^*ST-opt*^) accuracy of the 47,123 MMDB ligand conformer models to the corresponding experimental 3-D structure, before and after the conformer model clustering procedure, as a function of: **(a)** the non-hydrogen atom count, **(b)** the rotatable bond count, **(c)** the effective rotor count, and **(d)** the RMSD clustering threshold (*RMSD*^*cluster*^).

**Figure 11:**
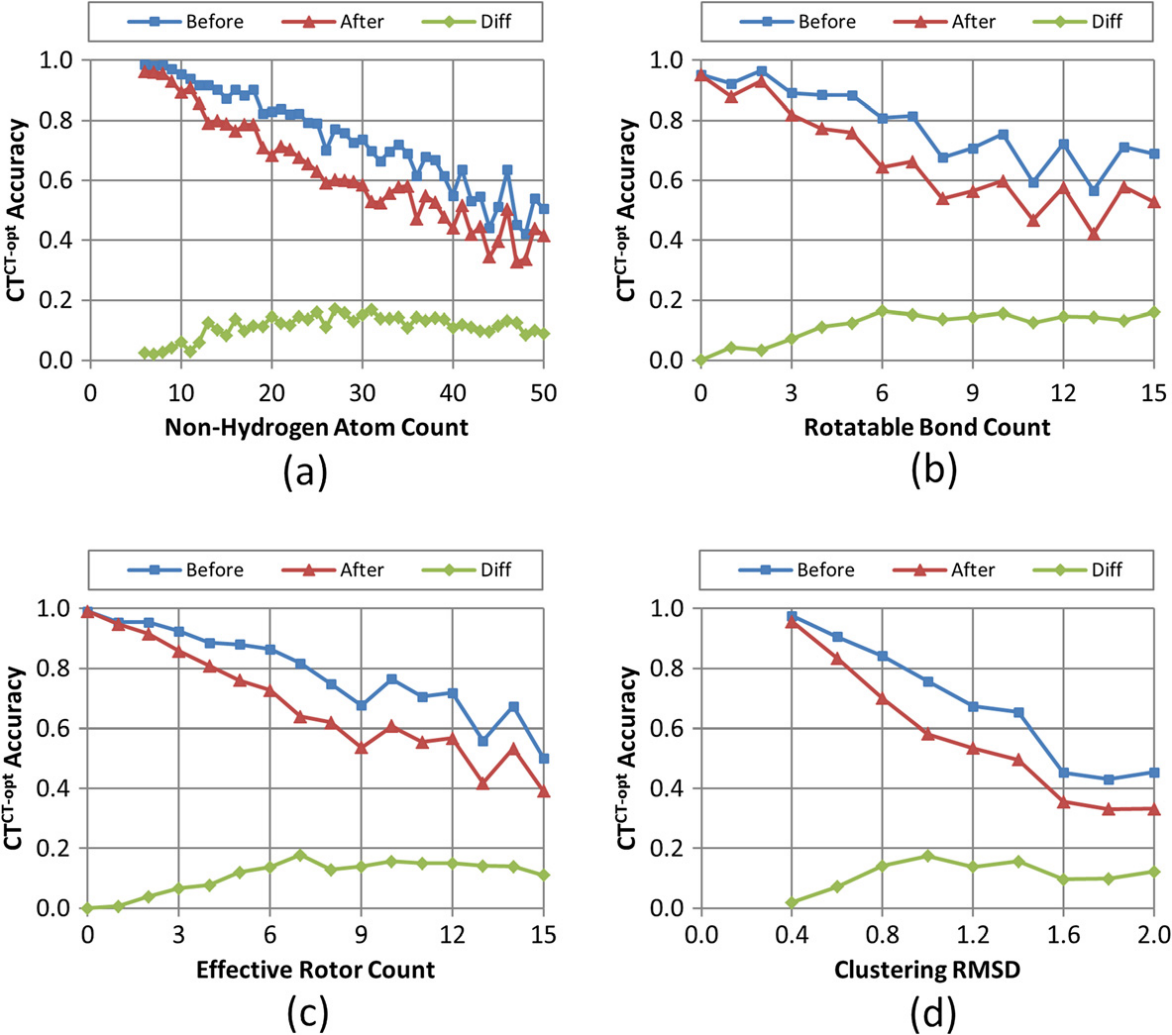
**Average C*T***^***CT-opt***^**accuracy as a function of the molecular size, flexibility, and *RMSD***^***cluster***^**.** The average conformer model color-optimized color-Tanimoto (*CT*^*CT-opt*^) accuracy of the 47,123 MMDB ligand conformer models to the corresponding experimental 3-D structure, before and after the conformer model clustering procedure, as a function of: **(a)** the non-hydrogen atom count, **(b)** the rotatable bond count, **(c)** the effective rotor count, and **(d)** the RMSD clustering threshold (*RMSD*^*cluster*^).

**Figure 12:**
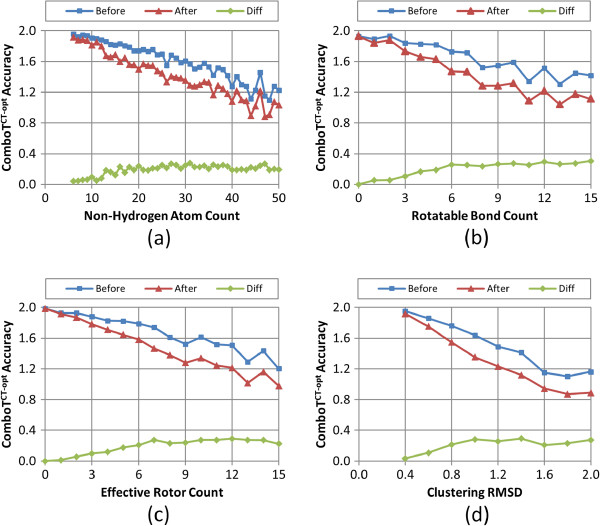
**Average *ComboT***^***CT-opt***^**accuracy as a function of the molecular size, flexibility, and *RMSD***^***cluster***^**.** The average conformer model color-optimized combo-Tanimoto (*ComboT*^*CT-opt*^) accuracy of the 47,123 MMDB ligand conformer models to the corresponding experimental 3-D structure, before and after the conformer model clustering procedure, as a function of: **(a)** the non-hydrogen atom count, **(b)** the rotatable bond count, **(c)** the effective rotor count, and **(d)** the RMSD clustering threshold (*RMSD*^*cluster*^).

Figures 8, 9, 10, 11, and 12 show the five different measures of the conformer ensemble accuracy used (*i.e.*, RMSD, *ST*^*ST-opt*^, *ComboT*^*ST-opt*^, *CT*^*CT-opt*^, and *ComboT*^*CT-opt*^) as a function of molecular size and flexibility. [A further breakdown of RMSD and *ST*^*ST-opt*^ values as a function of molecular size and flexibility and the correlation between RMSD and *ST*^*ST-opt*^ can be found in Additional file 2: Figures S1-S7]. The linear nature of these curves demonstrates a clear association of the average PubChem3D conformer model accuracy with molecular size and flexibility. Least-squares fitting to the form of “*y* = *a* + *bx*” for each data series in the plots from panel (d) in Figures 8, 9, 10, 11, and 12 is summarized in Table 2. The least-squares fitting was also performed for the other data series in panels (a-c) of Figures 8, 9, 10, 11, and 12, but reported in Additional file 3 for brevity. As the *RMSD*^*cluster*^ value (as well as *N*_*NHA*_, *N*_*R*_ and *N*_*ER*_) increases, all five conformer model accuracies *before* and *after* clustering linearly changed. With the notable exception of *N*_*R*_, all R^2^ values for these fits were greater than 0.90. In the case of *N*_*R*_, not taking into account the flexibility of rings reduces the R^2^ values to as low as 0.78. (In fact, the primary motivation of the development of *N*_*ER*_[30] was to account for “noise” in linear fits of *N*_*R*_ such as these, to properly account for molecules that are effectively more flexible than their rotatable bond count would suggest.) While all the RMSD and *ST*^*ST-opt*^ average accuracy measures did linearly correlate with the *RMSD*^*cluster*^ value (Figures 8 and 9). However, for the *ComboT*^*ST-opt*^, *CT*^*CT-opt*^, and *ComboT*^*CT-opt*^, the diffe-rence between *before* and *after* clustering accuracy values did not always linearly correlate with the *RMSD*^*cluster*^ value [namely: Figure 10, panels (a,d); Figure 11 panels (a-d); and Figure 12 panels (a,d)]. In the case of *CT*^*CT-opt*^, the average differences appear to plateau just below 0.2, suggesting that there may be some maximum error as a result of the PubChem conformer clustering procedure. Echoes of this appear to be present in the difference statistics for any 3-D similarity measure that involves the CT measure (*i.e.*, *ComboT*^*ST-opt*^, *CT*^*CT-opt*^, and *ComboT*^*CT-opt*^).

**Table 2:** **Linear behavior of average conformer model accuracy as a function of *RMSD ***^***cluster ***^**value**

**Accuracy measure**	**Data series**	***a***	***b***	***σ***_***a***_	***σ***_***b***_	***σ***_***y***_	***R***^***2***^
*RMSD*	Before	−0.06	0.63	0.087	0.061	0.11	0.93
	After	−0.07	0.90	0.083	0.059	0.11	0.97
	Difference	−0.01	0.27	0.045	0.032	0.06	0.90
*ST*^*ST-opt*^	Before	1.06	−0.15	0.020	0.014	0.03	0.94
	After	1.06	−0.21	0.025	0.018	0.03	0.95
	Difference	−0.00	0.06	0.012	0.009	0.02	0.85
*ComboT*^*ST-opt*^	Before	2.13	−0.53	0.057	0.040	0.07	0.96
	After	1.99	−0.59	0.088	0.062	0.11	0.92
	Difference	0.14	0.06	0.060	0.043	0.08	0.19
*CT*^*CT-opt*^	Before	1.10	−0.34	0.038	0.027	0.05	0.95
	After	1.01	−0.36	0.050	0.035	0.06	0.93
	Difference	0.09	0.02	0.036	0.025	0.05	0.07
*ComboT*^*CT-opt*^	Before	2.16	−0.54	0.058	0.041	0.07	0.96
	After	2.03	−0.60	0.091	0.064	0.12	0.92
	Difference	0.13	0.06	0.065	0.046	0.08	0.17

Results of linear least-squares fitting of the average conformer model accuracies *vs.* RMSD clustering threshold (*RMSD*^*cluster*^) to the form of “*y* = *a* + *bx*”. The sigma values (*σ*_*a*_, *σ*_*b*_, and *σ*_*y*_) correspond to the standard deviation of the fit to the predicted “*a*”, “*b*”, and “*y*” values. The “*x*” values are the discrete *RMSD*^*cluster*^ values and the “*y*” values are the corresponding average accuracy measures found in the data series from panel (d) in Figures 8, 9, 10, 11, and 12.

Taking this all into account, in general, conformer model clustering increases the conformer RMSD value and decreases the four 3-D Tanimoto values, indicating the reduced accuracy of the conformer ensemble due to conformer clustering. The difference between the ensemble accuracies before and after clustering increases with the values of *N*_*NHA*_, *N*_*R*_, and *N*_*ER*_, implying that the effects of conformer clustering become more noticeable in bigger and more flexible molecules, which is expected considering that the *RMSD*^*cluster*^ value gets larger [Equation (3)]. As compared in Figures 9 and 11, the average conformer *CT*^*CT-opt*^ accuracy values show a larger decrease upon clustering than the average conformer *ST*^*ST-opt*^ values, meaning that the conformer *CT*^*CT-opt*^ values are more sensitive to clustering than the conformer *ST*^*ST-opt*^ values. However, conformer clustering decreases the average *ComboT*^*ST-opt*^ and average *ComboT*^*CT-opt*^ values (Figures 10 and 12) in a similar amount, again showing the insensitiveness of the ComboT value to the optimization type. A similar insensitiveness of the ComboT value to the optimization type was also observed in our previous studies [9,11], in which the distribution of the *ComboT*^*ST-opt*^ scores between randomly selected conformers were found very similar to that of the *ComboT*^*CT-opt*^.

What does this all mean? The average loss of accuracy of PubChem3D conformer ensembles behaves in a predictable fashion, even after sampling, as a function of molecular size and flexibility across PubChem3D similarity measures. There is a linear degradation of accuracy to reproduce bioactive conformers both before and after sampling procedures. In general, there is a modest amount of degradation of accuracy to reproduce bioactivity as a part of this sampling procedure. Generally speaking, one expects the worst-case minimum accuracy of 90% of the PubChem3D ensembles to be (as stated previously from Figure 7) 0.75, 1.09, 0.43, and 1.13, in terms of *ST*^*ST-opt*^, *ComboT*^*ST-opt*^, *CT*^*CT-opt*^, and *ComboT*^*CT-opt*^, respectively. This expected minimum accuracy improves linearly as the molecule becomes smaller or less flexible.

One may ask “how good or how bad are these worst-case minimum accuracies?” To answer this question, it is necessary to determine an appropriate cut-off value for a “close” reproduction of the experimental structure, and our recent study [11], which studied the statistical significance of the ROCS-based similarity scores, provides some clues on an appropriate choice of the cut-off values. In this study [11], the ROCS-based 3-D similarity scores between randomly-selected biologically-tested compounds were computed, and from the distribution of these scores, conversion tables were generated which convert a ROCS-based similarity score to the *p*-value of getting that particular score by randomly selecting two biologically-tested conformers. According to these conversion tables, the *p*-value of getting a similarity score equal to the worse-case minimum accuracy by selecting two random conformers is 0.019, 0.002, 0.003, and 0.002 for *ST*^*ST-opt*^, *ComboT*^*ST-opt*^, *CT*^*CT-opt*^, and *ComboT*^*CT-opt*^, respectively. If the significance level (α) of 0.05 is employed, these *p*-values are small enough to reject the null hypothesis of getting a particular 3-D similarity score by chance. Although this interpretation also depends on the significance level one may choose, it is still true that these worst-case minimum accuracies of the conformer models (0.75, 1.09, 0.43, and 1.13, for *ST*^*ST-opt*^, *ComboT*^*ST-opt*^, *CT*^*CT-opt*^, and *ComboT*^*CT-opt*^, respectively) are much greater than one may expect from randomly selected conformer pairs (0.54 ± 0.10, 0.62 ± 0.13, 0.18 ± 0.06, and 0.59 ± 0.14, for *ST*^*ST-opt*^, *ComboT*^*ST-opt*^, *CT*^*CT-opt*^, and *ComboT*^*CT-opt*^, respectively) [9,11], implying structural similarity between the conformer model and the experimental structure. Also note that this interpretation is consistent with the fact that the 90% of the conformer models considered in this study have RMSD accuracies better than 1.1 Å, which is much tighter than the common upper bound (RMSD 2.0 Å) for successful reproduction of an experimental conformation in molecular docking, as mentioned above.

When it comes to biological activity data analysis, the present study shows that there will be a definitive upper limit to the PubChem3D conformer ensemble accuracy based on the molecular size and flexibility. While the results of the present study consider all sampled conformers, PubChem3D search and analysis tools use a diverse subset of sampled conformers, where the diverse subset selection criterion is the *ComboT*^*ST-opt*^ dissimilarity. [The reason for using the ComboT dissimilarity is that it considers both the ST and CT dissimilarity simultaneously. While the choice of the optimization type is somewhat arbitrary, our previous studies [9,11] have shown that the ComboT score is not very sensitive to the optimization type in the aggregate.] The effects of using a diverse set of sampled conformers will likely further decrease performance beyond that reported in this study. In addition, one can expect that, as the desired 3-D Tanimoto threshold increases in a given biological activity analysis, the ability to interrelate larger and more flexible molecules will decrease, not because they necessarily lack common biologically accessible conformer space, but because of the inherent similarity distance between the stored sampled conformers. This analysis also suggests that the use of a single “one-size-fits-all” similarity Tanimoto threshold for PubChem3D molecules may not be an ideal choice for conformer models sampled at different RMSD values. The results from this study suggest that conformer sampling may exacerbate the molecular size/flexibility dependency already present in conformer generation software [13]. Smaller and less flexible molecules in PubChem3D will have a tighter conformational sampling (with a smaller spacing between conformers) than larger and more flexible molecules, and therefore, can interrelate more molecules at a given Tanimoto value. In addition, smaller and less flexible molecules will have fewer sampled conformers in their respective conformer ensemble and will likely have less of a reduction in accuracy due to the use of a diverse subset. As a result, a search using a smaller and less flexible molecule as a query is likely to return more 3-D similar molecules than a search using a larger and more flexible query molecule. Furthermore, even if a large or flexible molecule is used as a 3-D similarity query, an increasing proportion of returned results are likely to be smaller or less flexible as the Tanimoto value is increased. This potential bias towards conformer models with smaller sampling distances may be worth further consideration and study to develop a more reliable 3-D similarity-based biological activity analysis method.

### Effects of experimental uncertainties upon conformer model accuracies

Like any experimentally-derived measurements, the crystal structures stored in PDB have uncertainties in their atomic coordinates, and the interpretation of the accuracy of a computationally-derived conformer model should take into account the positional uncertainty of the corresponding experimental ligand structure. For example, if the positional uncertainty in the experimental structure is greater than the RMSD value of the conformer model, comparison between the experimental and theoretical ligand structures are not particularly meaningful. The average positional errors in atoms in a crystal structure can be evaluated with the diffraction-component precision index (DPI) [41,42], which can be approximated as proposed by Blow [43], using information commonly contained in the header of a PDB file. In the study by Hawkins *et al.*[18], the crystal structures with the DPI of < 0.42 Å were considered to be precise enough for the use as a standard dataset for validation of conformer generators, and in this way, the conformer models with the RMSD value of > 0.6 Å (= √2 × DPI [44]) were guaranteed to be meaningful predictions.

**Figure 13:**
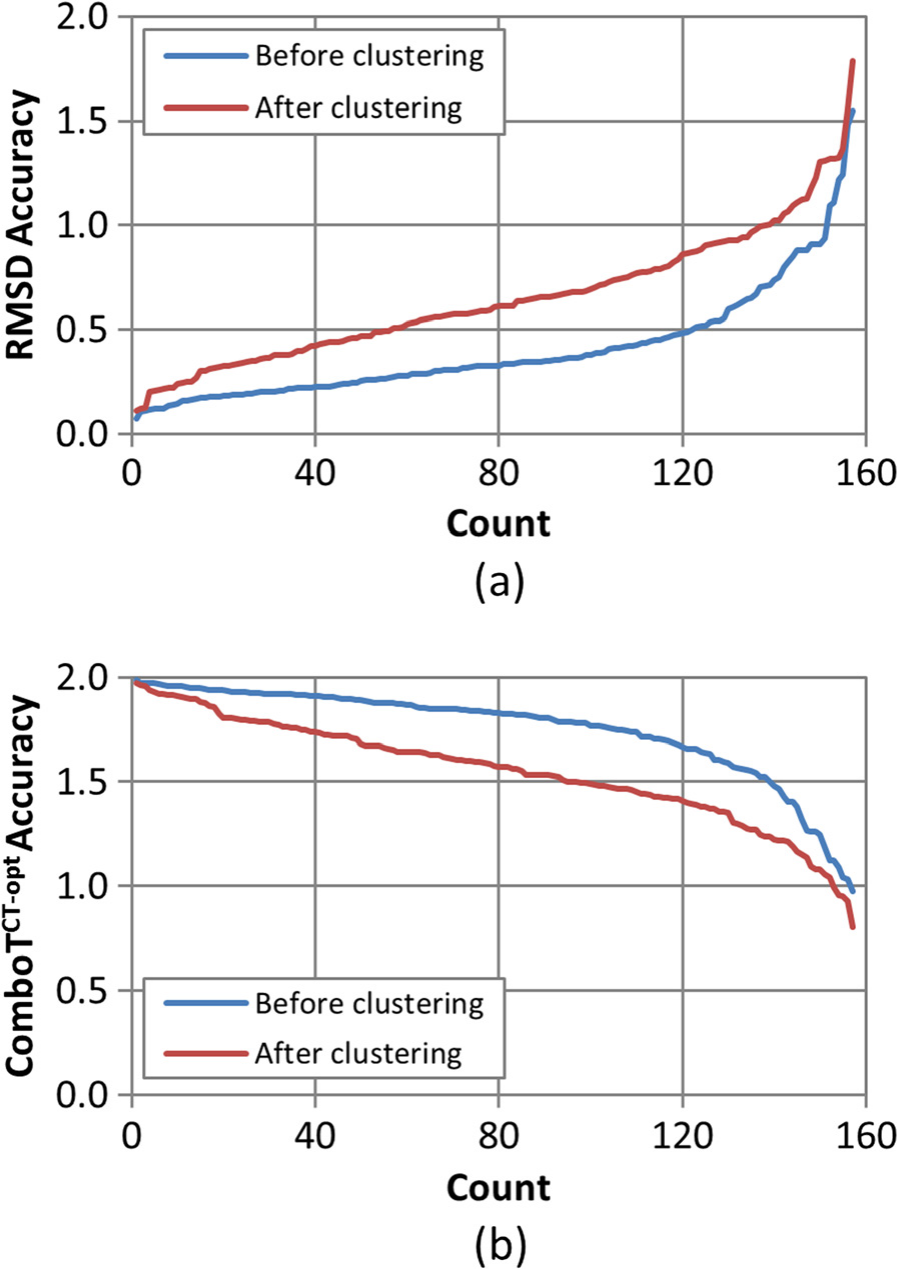
**Distribution of the conformer model accuracies for the 157 high-quality ligands.** Distribution of before- and after-clustering accuracies of the conformer models from the 157 ligand molecules selected from the 47,123 PDB ligands considered in the present study: **(a)** the RMSD accuracy, and **(b)** the *ComboT*^*CT-opt*^ accuracy.

Although the present study did not focus on potential issues concerning the experimental uncertainties of PDB structures [13,32-36], it is still valuable to test the conformer model accuracy against a set of highly-reliable experimental structures. Therefore, a set of 157 high-quality ligand molecules (Additional file 4) was constructed from a set of 197 PDB structures recommended in the study by Hawkins *et al*. [18] (see the Methods section). The distribution of the *RMSD* and *ComboT*^*CT-opt*^ accuracies for these 157 structures are shown in Figure 13, and their average and median values are compared in Table 3, with those from the 197 molecules considered in the study by Hawkins *et al*. [18] [The *ComboT*^*CT-opt*^ accuracy is identical to the Tanimoto Combo in their study]. As shown in Table 3, when going from the 47,123-ligand set to 157-ligand set, the average RMSD value of the conformer models increased by 0.08 Å (from 0.57 Å to 0.65 Å) and the average *ComboT*^*CT-opt*^ accuracy decreased by 0.06 (from 1.61 to 1.55). These differences do not seem very meaningful, considering the standard deviations for the *RMSD* and *ComboT*^*CT-opt*^ accuracies of the two sets.

**Table 3:** **Comparison of the average and median *RMSD *and *ComboT***^***CT-opt ***^**values between different PDB ligand sets**

**Accuracy measure**	**47,123 Ligands**		**157 Ligands**		**197 Ligands (Ref.**[18]**)**
	**Before clustering**	**After clustering**	**Before clustering**	**After clustering**	
*RMSD* (Å)	0.39 (±0.24) / 0.30	0.57 (±0.36) / 0.50	0.40 (±0.26) / 0.33	0.65 (±0.31) / 0.61	0.67 / 0.51
*ComboT*^*CT-opt*^	1.77 (±0.20) / 1.85	1.61 (±0.27) / 1.70	1.75 (±0.22) / 1.83	1.55 (±0.25) / 1.58	1.56 / 1.64

Numbers before and after slash are the mean and median, respectively, and numbers in parentheses are the standard deviations. The 47,123-ligand set are all the PDB ligand molecules considered in the present study, and the 157-ligand set contains 157 high-quality ligand molecules selected from the 47,123 compounds. The 197-ligand set contains 197 high-quality ligand molecules from the study by Hawkins *et al*. [18].

Note that the average RMSD value of the 157-ligand set differed only by 0.02 from that of the 197-ligand set from the study of Hawkins *et al.* (0.65 Å *vs*. 0.67 Å) [18]. The difference in the *ComboT*^*CT-opt*^ accuracy between the two sets were 0.01 (1.55 and 1.56 for the 157- and 197-ligand sets, respectively). Considering that our study used OMEGA parameters different from those used in their study, the conformer model accuracies from the two studies do not seem very different.

## Conclusion

In the present study, conformer ensembles for 47,123 PDB ligand molecules from MMDB were computationally generated using the PubChem3D approach. The accuracy of reproduction of the conformer models was investigated in comparison to the experimentally-derived structures as a function of the RMSD and the PubChem3D similarity scores (*i.e.*, *ST*^*ST-opt*^, *ComboT*^*ST-opt*^, *CT*^*CT-opt*^, and *ComboT*^*CT-opt*^). The PubChem3D conformer sampling procedure increased the RMSD value of the conformer ensemble by 0.18 ± 0.12 Å, and decreased the accuracy of the *ST*^*ST-opt*^, *ComboT*^*ST-opt*^, *CT*^*CT-opt*^, and *ComboT*^*CT-opt*^ accuracies by 0.04 ± 0.03, 0.16 ± 0.09, 0.09 ± 0.05, and 0.15 ± 0.09, respectively (see Table 1), indicating a decrease in the conformer ensemble accuracy in general. For all five accuracy measures (RMSD, *ST*^*ST-opt*^, *ComboT*^*ST-opt*^, *CT*^*CT-opt*^, and *ComboT*^*CT-opt*^), the conformer model accuracies before and after clustering linearly decreased with the increase in the *RMSD*^*cluster*^ value (as well as *N*_*NHA*_, *N*_*R*_ and *N*_*ER*_), with R^2^ values to fit these curves greater than 0.91 (see Figures 8, 9, 10, 11 and 12 and Table 2).

Whereas the change in the C*T*^*CT-opt*^ accuracy (0.09 ± 0.05) upon clustering was much greater than the *ST*^*ST-opt*^ average difference (0.04 ± 0.03), the *ComboT*^*ST-opt*^ and *ComboT*^*CT-opt*^ changes had similar average and standard deviations (0.16 ± 0.09 *vs.* 0.15 ± 0.09). This implies that, in general, while the *CT*^*CT-opt*^ accuracy is more sensitive to the clustering than the *ST*^*ST-opt*^ accuracy, the effect of the clustering upon the ComboT accuracy is not sensitive to the optimization type. Similarly, while the rate of the decrease of the *ST*^*ST-opt*^ accuracy with the increase in molecular size and flexibility was much slower than that of the *CT*^*CT-opt*^ accuracy (Figure 9*vs.* Figure 11), the *ComboT*^*ST-opt*^ and *ComboT*^*CT-opt*^ accuracies decreased at a similar rate (Figure 10 vs. Figure 12).

This study shows that there is a definitive limit in the ability of the PubChem3D sampled conformer models to reproduce the bioactive conformations found in PDB ligands. This study also suggests that larger and more flexible molecules may be less able to interrelate with other larger and more flexible molecules at a given Tanimoto value than smaller and less flexible molecules do. [This is also supported by our recent study [8] on the PubChem 3-D neighbors. The PubChem 3-D neighbors (also known as “similar conformers”) are defined as any two compounds that are structurally similar (with *ST*^*ST-opt*^ ≥ 0.8 and *CT*^*ST-opt*^ ≥ 0.5), and it was found that compounds without 3-D neighbors occur more frequently among larger compounds than among smaller compounds. In addition, smaller molecules tend to have more 3-D neighbors than larger molecules]. As a result, one may want to consider such effects when performing a 3-D similarity search or 3-D biological activity data analysis. Specifically in the case of 90% of the PubChem3D conformer models, in general, one can expect the worst-case minimum accuracy to be 0.75, 1.09, 0.43, and 1.13, in terms of *ST*^*ST-opt*^, *ComboT*^*ST-opt*^, *CT*^*CT-opt*^, and *ComboT*^*CT-opt*^, respectively (see Figure 7). These values are expected to linearly improve as the molecules considered become smaller and less flexible. In addition, these values may become worse if a diverse subset of sampled conformers is used.

## Methods

### Datasets

The experimental 3-D structures of small molecules were downloaded from the Molecular Modeling Database (MMDB) ligand dataset [45,46] as available from the PubChem Substance database at the National Center for Biotechnology Information (NCBI) (as of July 1, 2010). Ligands too small or too big were discarded by limiting the non-hydrogen atom count to 6 – 50. Ligands too flexible (with an effective rotor count greater than 15) were also eliminated. This filtering stage resulted in a set of 47,123 3-D non-unique, organic (*i.e.*, carbon containing) 3-D experimental reference structures, where a 3-D conformer model could be generated. The distributions of molecular size and flexibility of the dataset are depicted in Figure 1.

To test effects of the experimental uncertainties upon the evaluation of the conformer model accuracies, a subset of the 47,123-ligand set, which contains 157 “high-quality” ligand structures, was constructed in the procedures described below.

(1) Select the PubChem Substance records associated with the MMDB records for the 197 PDB structures determined in the study by Hawkins *et al*. [18]. These PDB structures were determined by considering the local quality of fit of the ligand to its density, as well as global level metrics of the protein structure. Because some of the 197 PDB structures had multiple ligands, there were 265 PubChem Substance records associated with these protein structures. Note that, because their study provided a list of the PDB identifiers (without a unique ligand identifier), it was difficult to determine what ligands were actually included in the 197-ligand set. Therefore, next filtering steps similar to those used in their study were taken subsequently.

(2) Select the PubChem Substance records that are neither too rigid nor too flexible (3 ≤ *N*_*R*_ ≤ 16), and that are neither too small nor too large (8≤ *N*_*NHA*_ ≤ 50). This filtering stage resulted in 200 PubChem Substance records.

(3) Select the PubChem Substance records with good “local” quality of fit to the density. Hawkins *et al.*[18] used three metrics for this purpose: the real-space correlation coefficient (RSCC) [47], the real-space R-value (RSR) [48], and the occupancy-weighted B-factor (OWAB). In the present study, the same criteria as used in their study (RSCC > 0.9, RSR < 0.2, and 1 < OWAB < 50) were applied, after downloading these data from the electron density server (EDS) [49,50]. After this step, 176 structures were remained.

(4) Some of the remaining 176 PubChem Substance Records were associated with identical PubChem Compound Records, or had the same three-letter PDB ligand codes, implying that they were the same ligand molecule. In these cases, the one with the largest RSCC value was retained, and the others were removed. After removing the redundancy, there were 164 structures remained.

(5) When any pair of the remaining 164 structures had the PubChem 2-D similarity score of > 0.9 (computed using the PubChem 2-D subgraph fingerprints [5] and the Tanimoto equation [6,7]), the one with the largest RSCC value was retained and the other was removed. [In the study of Hawkins *et al*. [18], the LINGOS method [51] was used instead of the PubChem fingerprint to remove too similar molecules.] There were 157 ligands remained after this final filtering stage.

### Conformer generation using OMEGA

The conformer ensemble for each molecule in the dataset was generated using the OMEGA software [28] from the OpenEye Scientific Software, Inc. The OMEGA application performs conformer generation in two primary stages: fragment generation and torsion driving. The fragment generation stage splits the input structure into smaller pieces that are energy minimized and conformationally sampled to get diverse 3-D representations for each molecule fragment. The torsion driving stage reassembles and iterates over the fragments from the first stage using particular rule-based torsion angles that depend on the molecular environment between connecting fragments. More detailed description of the OMEGA software is given elsewhere [18,52].

OMEGA has many adjustable parameters to generate conformations with particular attributes, and the optimal set of parameter values used for the present study was based on our recent study [13]. The Merck Molecular Force Field (MMFF94s) without coulombic terms (MMFF94s_NoEstat) was used with the "startfact" value of 20. The energy window value of 25 kcal/mol was employed for both model building and torsion driving stages. The values used for other parameters were identical to those used in the previous study [13].

As pointed out in a recent review by Scior *et al.*[53], because adequate conformational space coverage is an important requirement for reliable 3-D similarity computations, it would be desirable to consider as many conformations per molecule as possible. However, because it would require tremendous computational resources, it is inevitable to find a compromise between computational cost and conformational coverage. The PubChem3D conformer generation procedure generates a maximum of 100,000 conformers for each chemical structure. As demonstrated in our previous study [13], this limit may not be enough for very flexible and large molecules, resulting in truncation of conformational search. However, in the same study [13], it was shown that the 100-K limit does not cause a noticeable decrease in the “average” conformer model accuracy for smaller and less flexible molecules (*i.e.*, with *N*_*NHA*_≤35 and *N*_*ER*_≤15). Therefore, this 100-K limit seems adequate for these molecules in general.

### Clustering of conformer ensembles

After conformer models were produced, a data reduction was performed whereby conformers were sampled to identify a random set of conformers that have a minimum RMSD distance to each other. This minimum RMSD distance was determined by rounding the *RMSD*^*pred*^ value [in Equation (3)] to the nearest 0.2 increment *i.e.*, Equation (4)]. The conformers in each conformer ensemble were down-sampled using a partition-based clustering scheme, as described in our previous study [15], with the RMSD as a distance threshold between conformers (that is, *RMSD*^*cluster*^) and the lowest-energy conformer in each partition as an initial “seed” structure for clustering of that partition. The centroid of each cluster was selected as the representative conformer of that cluster to construct a smaller conformer model with 500 conformers or less. If the conformer model had more than 500 conformers after sampling, it was re-clustered with the *RMSD*^*cluster*^ value incremented by a further 0.2. This re-clustering process was repeated as many times as necessary to reduce the overall conformer count to be 500 or less. Note that, because the lowest-energy conformer in each partition was used as an initial seed, low-energy conformers are more likely to be included than high-energy conformers when all partitions are combined together for next round of clustering. As a result, the final conformer model sampled though clustering is more likely to include low-energy conformers than high-energy conformers. All RMSD values computed in this study used the OEChem OERMSD function with: “overlay” turned on to allow rotation/translation to yield the lowest possible RMSD value; and “automorph” detection turned on to allow proper treatment of symmetrically equivalent atoms, except when its use resulted in excessive run-time [an extremely rare event (at a rate of about 1 in 10,000) generally caused by large, nearly symmetric molecules].

### Evaluation of ensemble accuracies

The accuracy of the clustered ensembles was estimated using five different accuracy measures: RMSD, *ST*^*ST-opt*^, *ComboT*^*ST-opt*^, *CT*^*CT-opt*^, and *ComboT*^*CT-opt*^. The latter four accuracy measures were computed using ROCS [37,38,52]. Note that the generated conformer model had up to 500 conformers, and the accuracy of the conformer model was evaluated by selecting the best conformer that was closest to the experimental structure (that is, the one with the smallest RMSD value or the largest ROCS-based similarity values).

## Competing interests

The authors declare that they have no competing interests.

## Authors’ contributions

EEB generated the data. SK wrote the first manuscript. Both EEB and SK analyzed the data, and SHB reviewed the final manuscript. All authors read and approved the final manuscript.

## Supplementary Material

Additional file 1 **List of the 47,123 small-molecule ligands considered in the study.** This file contains a list of the PC Substance ID for small molecules considered in this study. Click here for file

Additional file 2 **Distribution of the conformer ensemble accuracies.** This file contains figures that show the distributions of the RMSD and the *ST*^*ST-opt*^ accuracies of the conformer models as a function of *N*_*NHA*_, *N*_*R*_, and *N*_*ER*_ (Additional file 2: Figures S1-S6) and correlation between the two accuracy measures (Additional file 2: Figure S7). Click here for file

Additional file 3 **Least-squares fitting of the plots of conformer model accuracy*vs***. ***N***_***NHA***_, ***N***_***R***_, ***N***_***ER***_, **and*RMSD***^***cluster***^**.** This file contains results of linear least-squares fitting of the plots (in Figures 8, 9, 10, 11, and 12) of the average conformer model accuracies *vs.* each of three independent variables [*i.e.*, the non-hydrogen atom count (*N*_*NHA*_), the rotatable bond count (*N*_*R*_), and the effective rotor count (*N*_*ER*_)] to the form of *y* = *a* + *bx*. Click here for file

Additional file 4 **List of the 157 high-quality ligands selected from the 47,123 ligands.** This file contains a list of the PC Substance ID for 157 high-quality ligands selected from the 47,123 molecules. Click here for file
