# Supplementary material for: PubChem3D: conformer ensemble accuracy
Source: J Cheminform. 2013 Jan 7;5:1. doi: 10.1186/1758-2946-5-1 (PMC3547714; doi:10.1186/1758-2946-5-1)
Supplement: Additional file 2 — Distribution of the conformer ensemble accuracies. This file contains figures that show the distributions of the RMSD and the STST-opt accuracies of the conformer models as a function of NNHA, NR, and NER (Additional file 2: Figures S1-S6) and correlation between the two accuracy measures (Additional file 2: Figure S7). [file 1758-2946-5-1-S2.pdf]

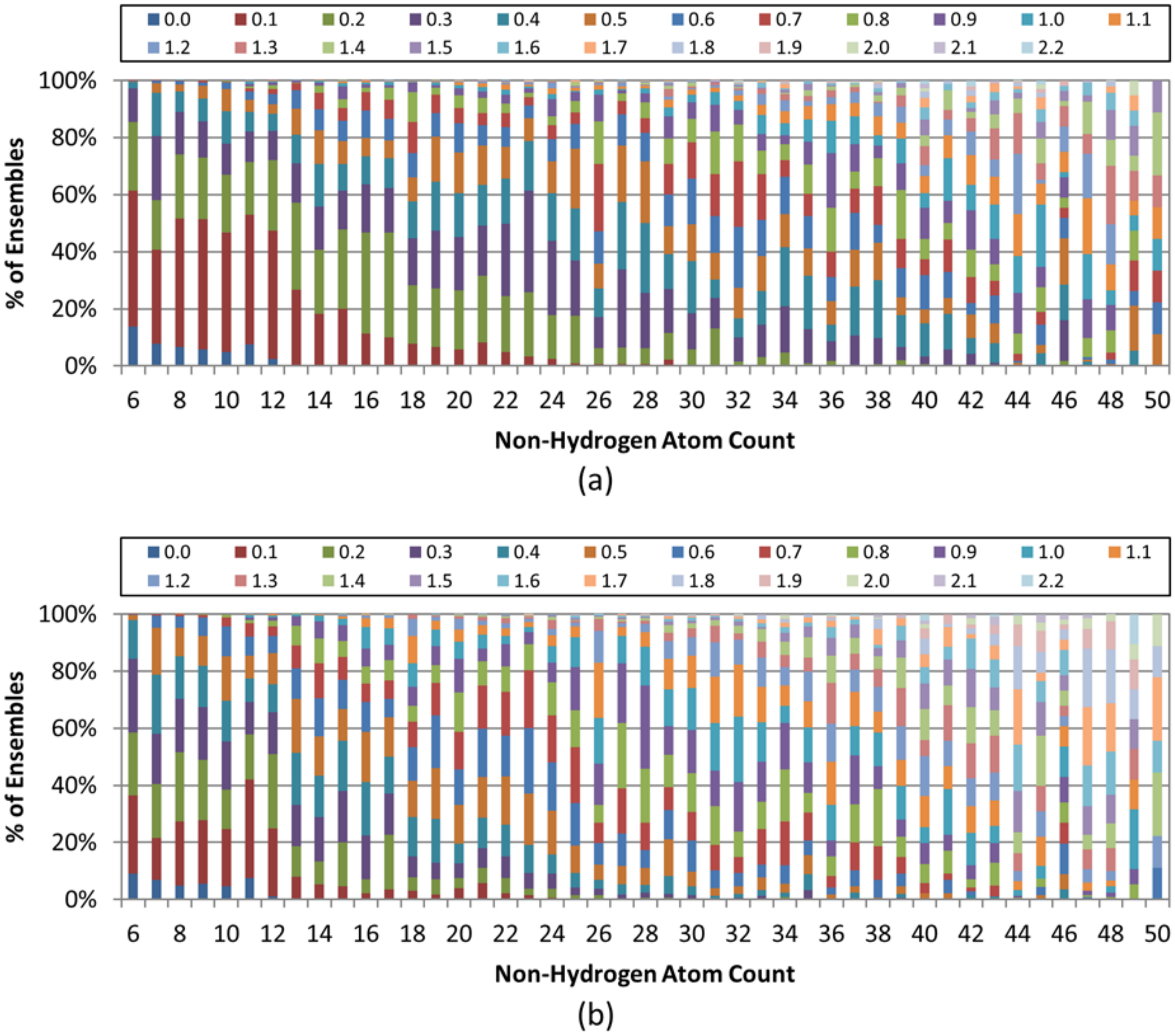

Figure S1. Distribution of the RMSD accuracy of the conformer model vs. the non-hydrogen atom count. The % distribution of the RMSD accuracy of the conformer model as a function of the non-hydrogen atom count: (a) before clustering and (b) after clustering.

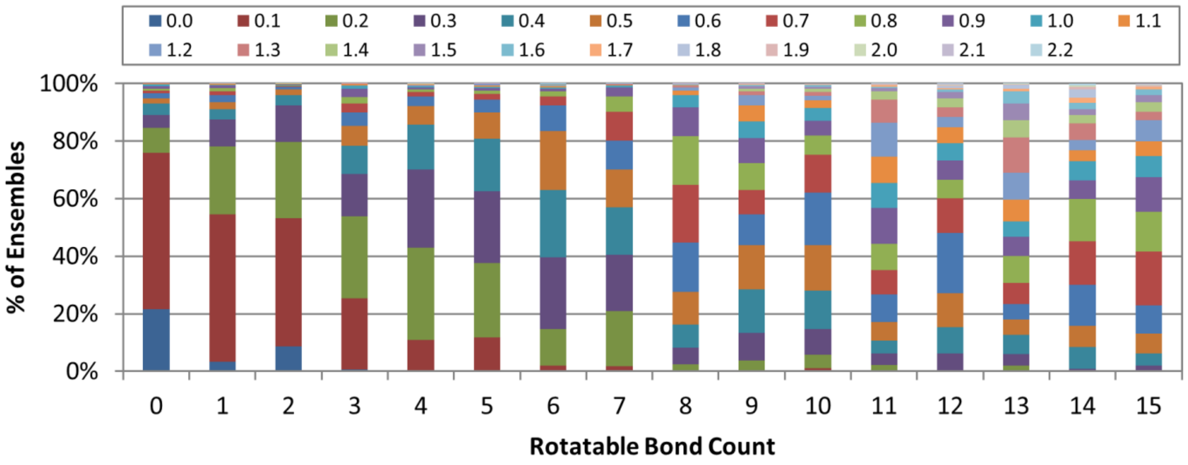

(a)

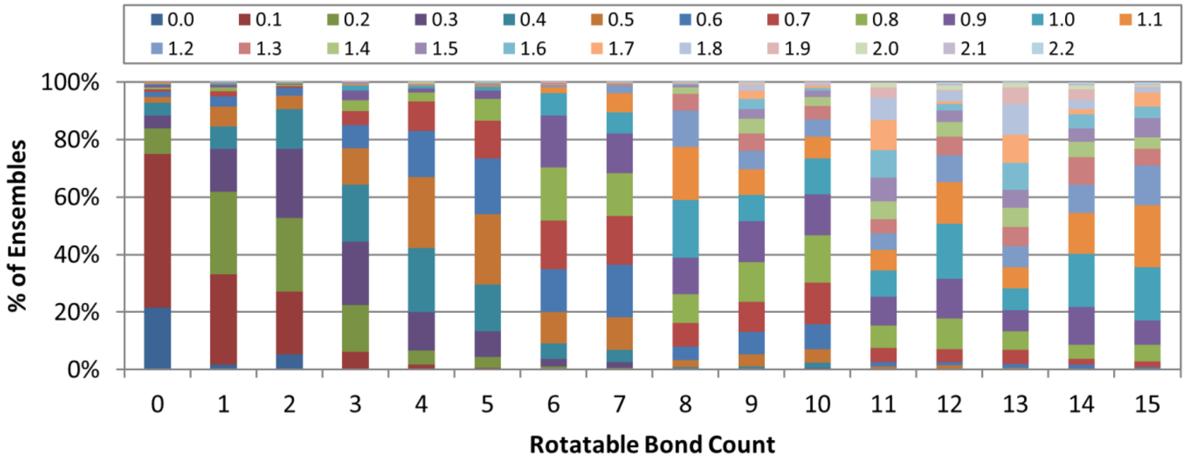

(b)

Figure S2. Distribution of the RMSD accuracy of the conformer model vs. the rotatable bond count. The % distribution of the RMSD accuracy of the conformer model as a function of the rotatable bond count: (a) before clustering and (b) after clustering.

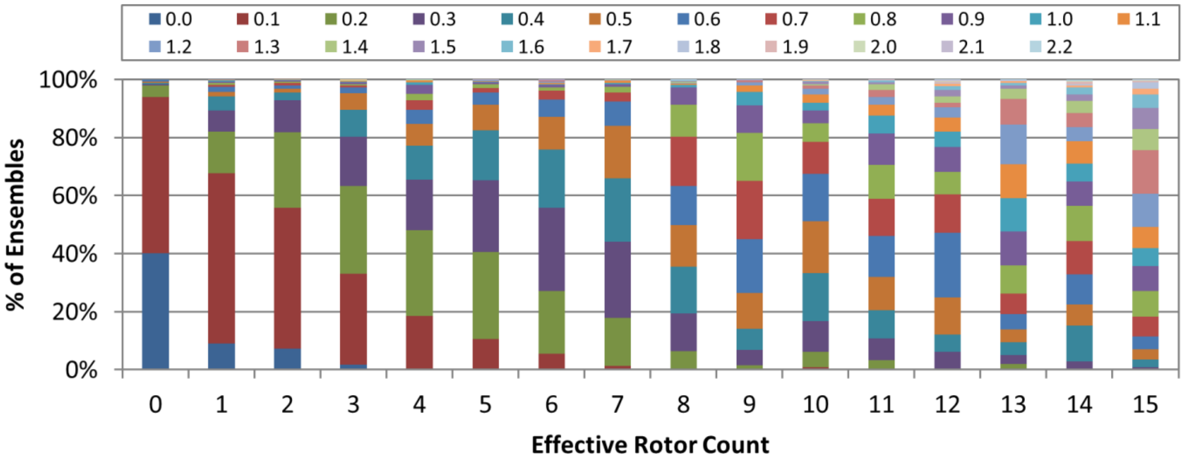

(a)

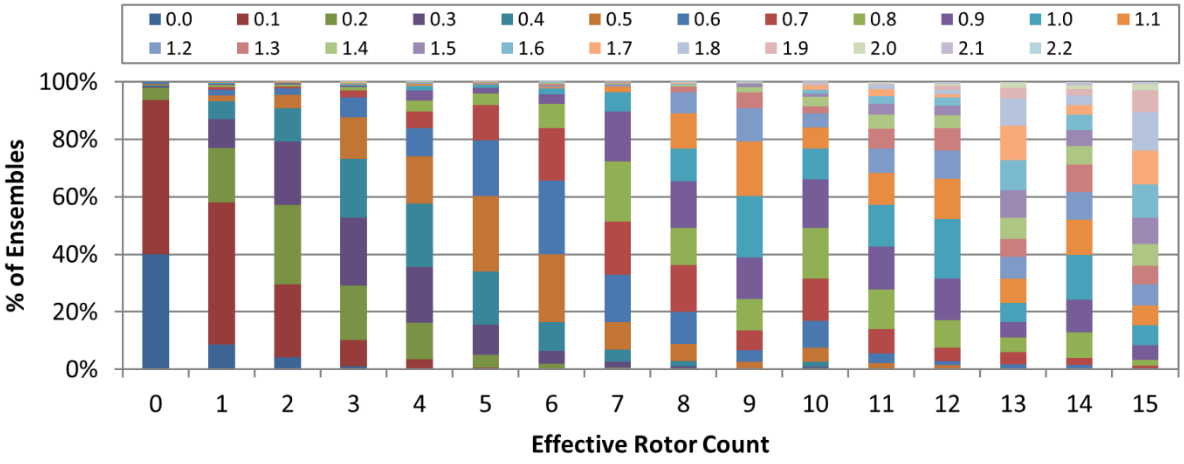

(b)

Figure S3. Distribution of the RMSD accuracy of the conformer model vs. the effective rotor count. The % distribution of the RMSD accuracy of the conformer model as a function of the effective rotor count: (a) before clustering and (b) after clustering.

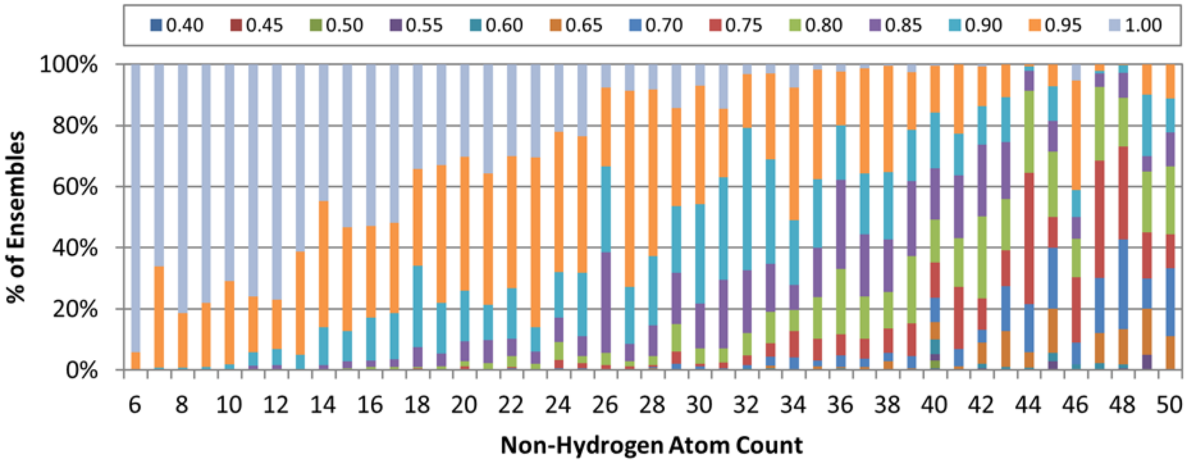

(a)

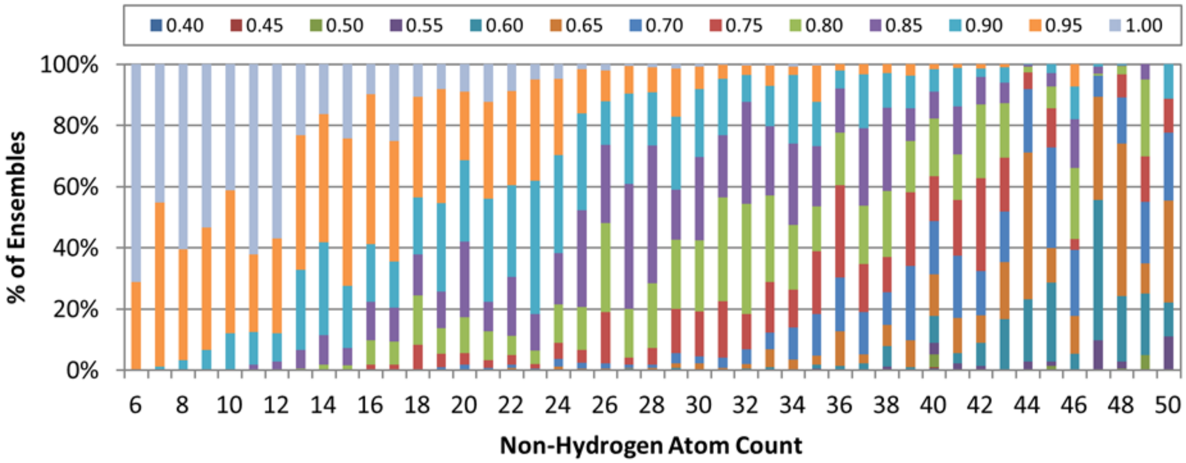

(b)

Figure S4. Distribution of the shape-optimzed shape-Tanimoto ($ST^{ST\text{-}opt}$) accuracy of the conformer model vs. the non-hydrogen atom count. The % distribution of the $ST^{ST\text{-}opt}$ accuracy of the conformer model as a function of the non-hydrogen atom count: (a) before clustering and (b) after clustering.

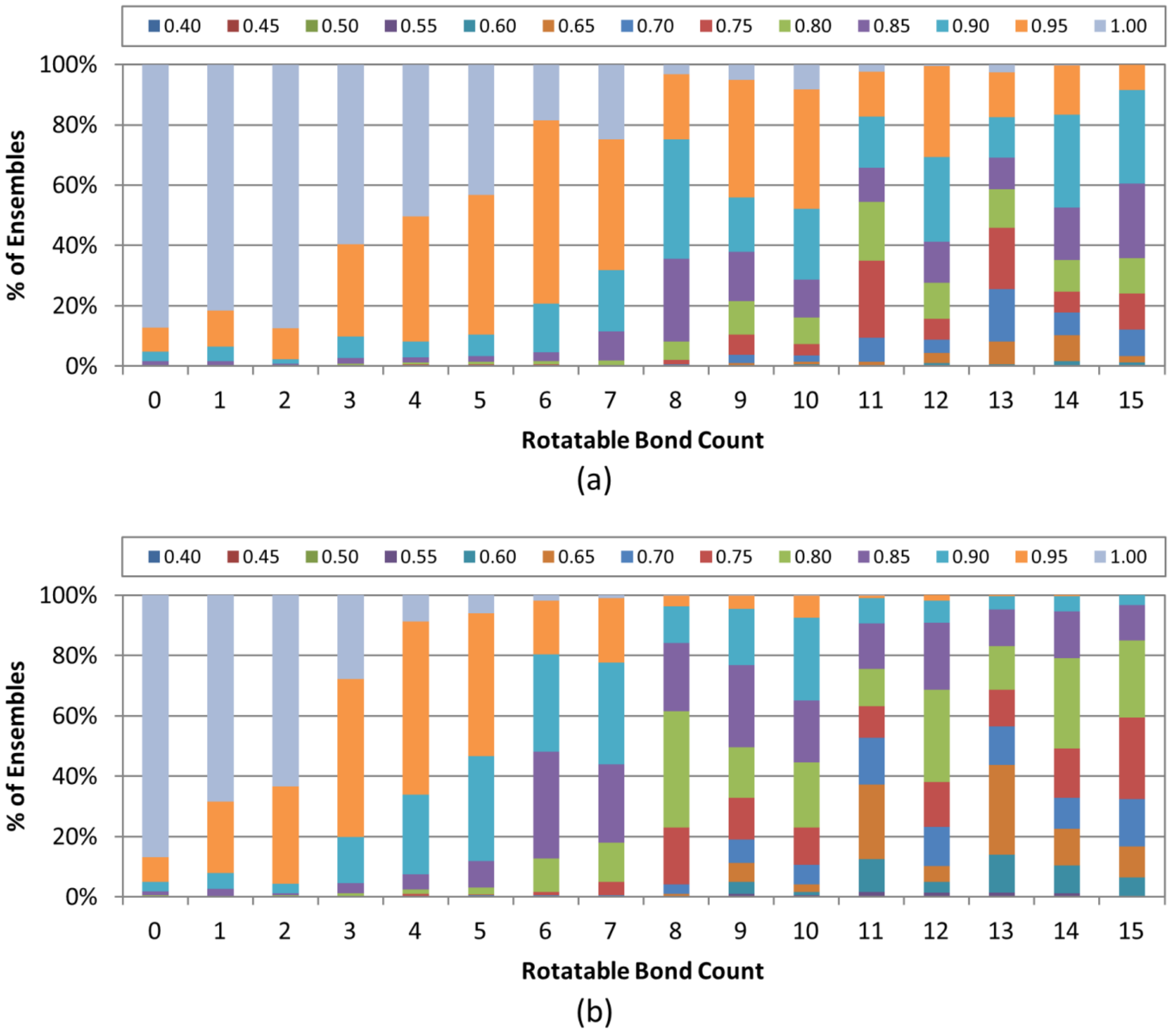

Figure S5. Distribution of the shape-optimzed shape-Tanimoto (*$ST^{ST\text{-}opt}$*) accuracy of the conformer model vs. the rotatable bond count. The % distribution of the $ST^{ST\text{-}opt}$ accuracy of the conformer model as a function of the rotatable bond count: (a) before clustering and (b) after clustering.

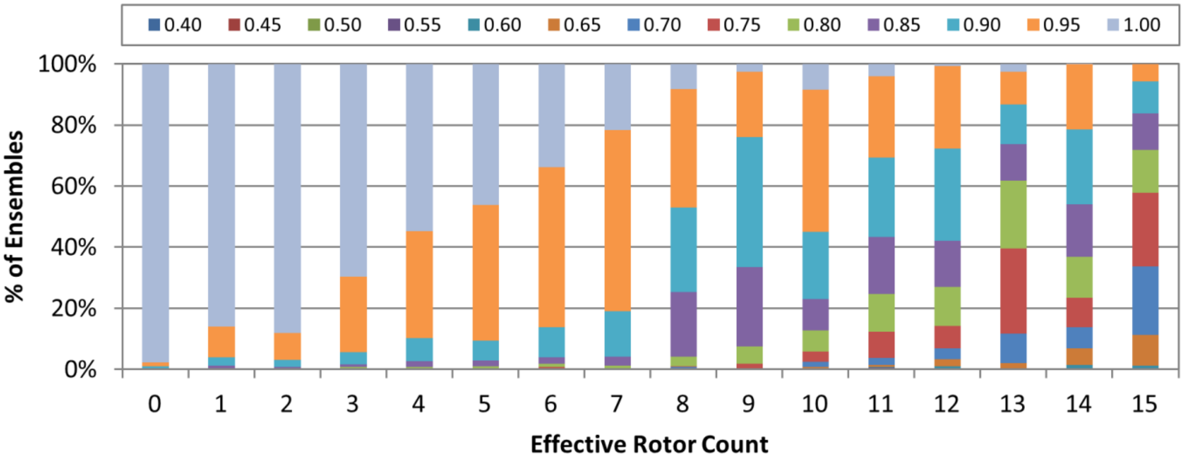

(a)

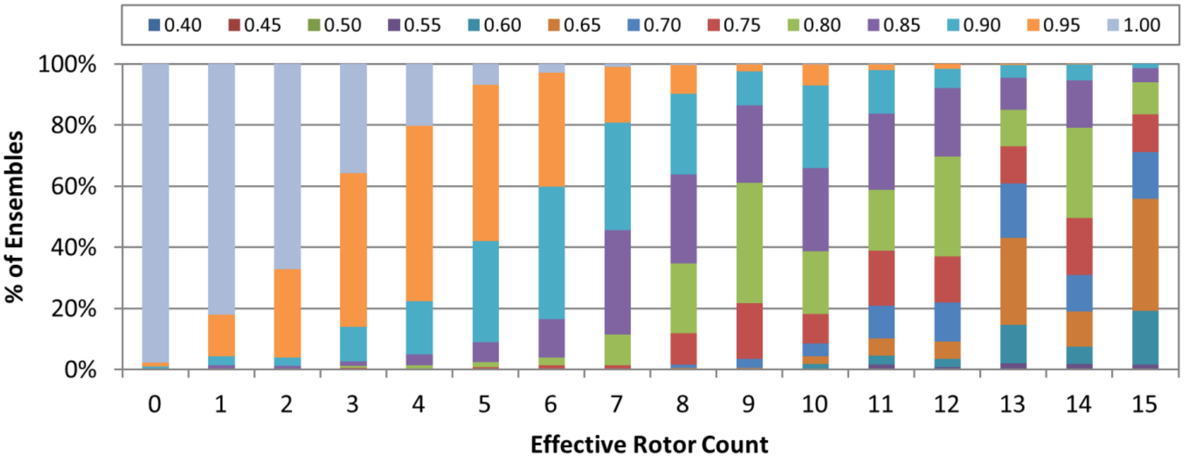

(b)

Figure S6. Distribution of the shape-optimzed shape-Tanimoto ($ST^{ST\text{-}opt}$) accuracy of the conformer model vs. the effective rotor count. The % distribution of the $ST^{ST\text{-}opt}$ accuracy of the conformer model as a function of the effective rotor count: (a) before clustering and (b) after clustering.

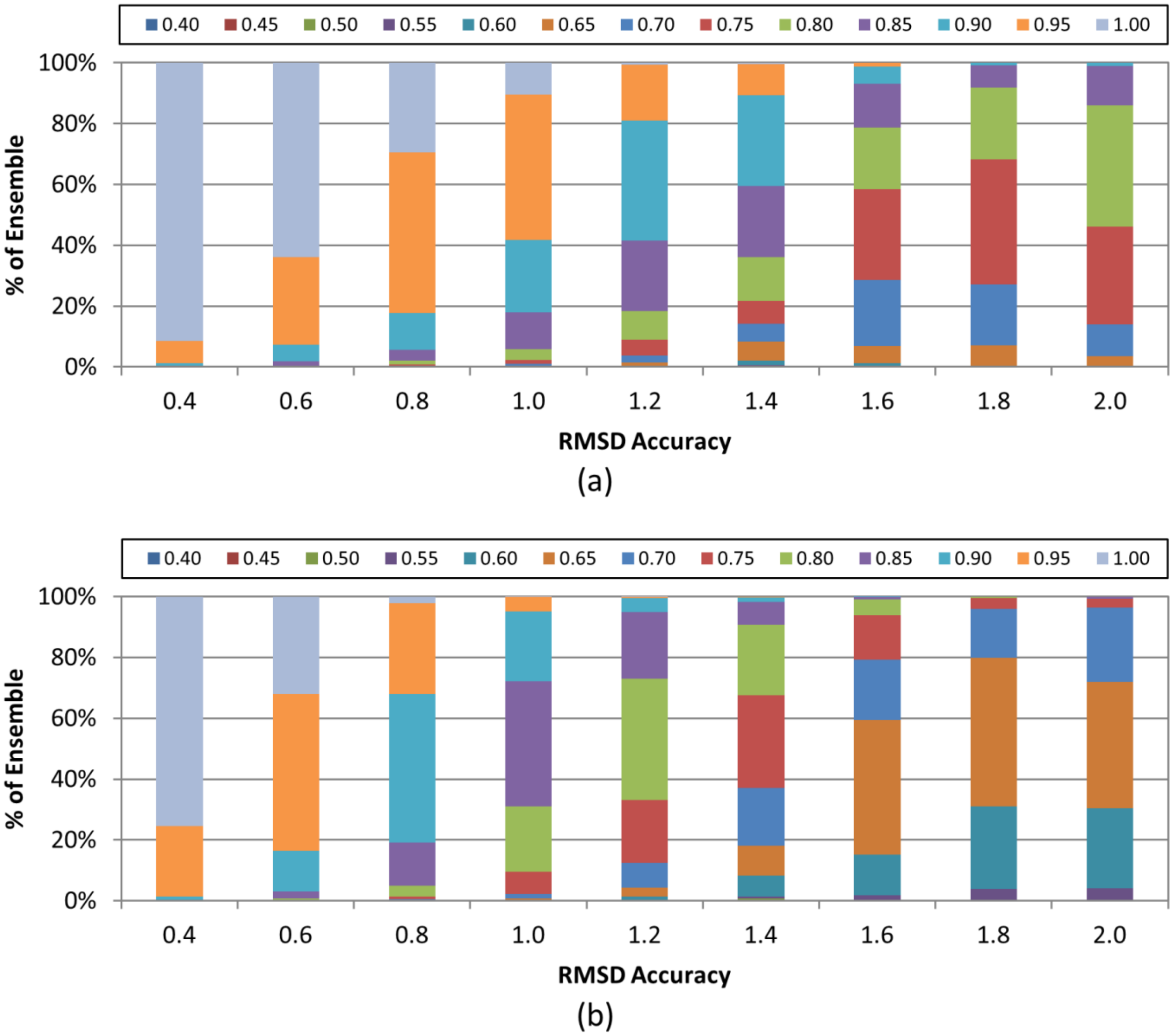

Figure S7. Correlation between the RMSD accuracy and shape-optimized shape-Tanimoto ($ST^{ST\text{-}opt}$) accuracy of the conformer models. The % distribution of the conformer models as a function of the $ST^{ST\text{-}opt}$ accuracy for a given RMSD accuracy: (a) before cluster and (b) after clustering.
