# Supplementary material for: PubChem3D: conformer ensemble accuracy
Source: J Cheminform. 2013 Jan 7;5:1. doi: 10.1186/1758-2946-5-1 (PMC3547714; doi:10.1186/1758-2946-5-1)
Supplement: Additional file 3 — Least-squares fitting of the plots of conformer model accuracyvs. NNHA, NR, NER, andRMSDcluster. This file contains results of linear least-squares fitting of the plots (in Figures 8, 9, 10, 11, and 12) of the average conformer model accuracies vs. each of three independent variables [i.e., the non-hydrogen atom count (NNHA), the rotatable bond count (NR), and the effective rotor count (NER)] to the form of y = a + bx. [file 1758-2946-5-1-S3.pdf]

Table S1. Linear behavior of average RMSD accuracy.

		All data points					
x	Data series	a	b	σ_a	σ_b	σ_y	R^2
N_{NHA}	Before	-0.0326	0.0226	0.0303	0.0010	0.09	0.92
	After	0.0142	0.0303	0.0334	0.0011	0.09	0.95
	Difference	0.0468	0.0077	0.0158	0.0005	0.04	0.84
N_R	Before	0.1126	0.0581	0.0357	0.0041	0.07	0.94
	After	0.1938	0.0802	0.0529	0.0060	0.11	0.93
	Difference	0.0812	0.0221	0.0306	0.0035	0.06	0.74
N_{ER}	Before	0.0498	0.0630	0.0291	0.0033	0.06	0.96
	After	0.0927	0.0890	0.0298	0.0034	0.06	0.98
	Difference	0.0429	0.0260	0.0233	0.0026	0.05	0.87

Results of linear least-squares fitting of the average RMSD conformer model accuracies to the form of "$y = a + bx$". The sigma values (σ_a, σ_b, σ_y) correspond to the standard deviation of the fit to the predicted "a", "b", and "y" values. The "y" values are the RMSD accuracy and the "x" values are the corresponding non-hydrogen atom count (N_{NHA}), the rotatable bond count (N_R), and the effective rotor count (N_{ER}) found for the data series in **Figure 8**, panels **(a-c)**.

Table S2. Linear behavior of average $ST^{ST\text{-}opt}$ accuracy.

		All data points					
x	Data series	a	b	σ_a	σ_b	σ_y	R^2
N_{NHA}	Before	1.0507	-0.0054	0.0082	0.0003	0.02	0.91
	After	1.0456	-0.0073	0.0086	0.0003	0.02	0.94
	Difference	0.0051	0.0020	0.0040	0.0001	0.01	0.84
N_R	Before	1.0098	-0.0129	0.0097	0.0011	0.02	0.91
	After	0.9991	-0.0191	0.0123	0.0014	0.03	0.93
	Difference	0.0107	0.0062	0.0058	0.0007	0.01	0.86
N_{ER}	Before	1.0219	-0.0139	0.0095	0.0011	0.02	0.92
	After	1.0178	-0.0206	0.0088	0.0010	0.02	0.97
	Difference	0.0040	0.0067	0.0047	0.0005	0.01	0.92

Results of linear least-squares fitting of the average RMSD conformer model accuracies to the form of "$y = a + bx$". The sigma values (σ_a, σ_b, σ_y) correspond to the standard deviation of the fit to the predicted "a", "b", and "y" values. The "y" values are the average $ST^{ST\text{-}opt}$ accuracy and the "x" values are the corresponding non-hydrogen atom count (N_{NHA}), the rotatable bond count (N_R), and the effective rotor count (N_{ER}) found for the data series in **Figure 9**, panels **(a-c)**.

Table S3. Linear behavior of average *ComboT*$^{ST\text{-}opt}$ accuracy.

x	Data series	All data points					
		a	b	σ_a	σ_b	σ_y	R^2
N_{NHA}	Before	2.0723	-0.0179	0.0235	0.0008	0.07	0.93
	After	1.9441	-0.0204	0.0245	0.0008	0.07	0.94
	Difference	0.1282	0.0026	0.0179	0.0006	0.05	0.31
N_R	Before	1.9505	-0.0449	0.0332	0.0038	0.07	0.91
	After	1.8726	-0.0632	0.0364	0.0041	0.08	0.94
	Difference	0.0779	0.0183	0.0221	0.0025	0.05	0.79
N_{ER}	Before	2.0050	-0.0497	0.0268	0.0030	0.06	0.95
	After	1.9423	-0.0682	0.0234	0.0027	0.05	0.98
	Difference	0.0627	0.0185	0.0264	0.0030	0.06	0.73

Results of linear least-squares fitting of the average RMSD conformer model accuracies to the form of "$y = a + bx$". The sigma values (σ_a, σ_b, σ_y) correspond to the standard deviation of the fit to the predicted "a", "b", and "y" values. The "y" values are the average *ComboT*$^{ST\text{-}opt}$ accuracy and the "x" values are the corresponding non-hydrogen atom count (N_{NHA}), the rotatable bond count (N_R), and the effective rotor count (N_{ER}) found for the data series in **Figure 9**, panels **(a-c)**.

Table S4. Linear behavior of average $CT^{CT\text{-}opt}$ accuracy.

		All data points					
x	Data series	a	b	σ_a	σ_b	σ_y	R^2
N_{NHA}	Before	1.0763	-0.0120	0.0138	0.0004	0.04	0.94
	After	1.0008	-0.0133	0.0159	0.0005	0.04	0.94
	Difference	0.0755	0.0013	0.0125	0.0004	0.04	0.20
N_R	Before	0.9572	-0.0231	0.0291	0.0033	0.06	0.78
	After	0.9033	-0.0313	0.0340	0.0039	0.07	0.82
	Difference	0.0538	0.0083	0.0155	0.0018	0.03	0.61
N_{ER}	Before	1.0054	-0.0289	0.0217	0.0025	0.05	0.91
	After	0.9620	-0.0376	0.0228	0.0026	0.05	0.94
	Difference	0.0434	0.0087	0.0176	0.0020	0.04	0.58

Results of linear least-squares fitting of the average RMSD conformer model accuracies to the form of "$y = a + bx$". The sigma values (σ_a, σ_b, σ_y) correspond to the standard deviation of the fit to the predicted "a", "b", and "y" values. The "y" values are the average $CT^{CT\text{-}opt}$ accuracy and the "x" values are the corresponding non-hydrogen atom count (N_{NHA}), the rotatable bond count (N_R), and the effective rotor count (N_{ER}) found for the data series in **Figure 11**, panels **(a-c)**.

Table S5. Linear behavior of average *ComboT*$^{CT\text{-}opt}$ accuracy.

x	Data series	All data points a	b	σ_a	σ_b	σ_y	R^2
N_{NHA}	Before	2.1075	-0.0182	0.0233	0.0008	0.07	0.93
	After	2.0035	-0.0213	0.0243	0.0008	0.07	0.94
	Difference	0.1040	0.0031	0.0176	0.0006	0.05	0.41
N_R	Before	1.9634	-0.0420	0.0365	0.0041	0.08	0.88
	After	1.8972	-0.0603	0.0400	0.0045	0.08	0.93
	Difference	0.0662	0.0183	0.0208	0.0024	0.04	0.81
N_{ER}	Before	2.0221	-0.0480	0.0297	0.0034	0.06	0.94
	After	1.9710	-0.0664	0.0258	0.0029	0.05	0.97
	Difference	0.0511	0.0184	0.0240	0.0027	0.05	0.77

Results of linear least-squares fitting of the average RMSD conformer model accuracies to the form of "$y = a + bx$". The sigma values (σ_a, σ_b, σ_y) correspond to the standard deviation of the fit to the predicted "a", "b", and "y" values. The "y" values are the average *ComboT*$^{CT\text{-}opt}$ accuracy and the "x" values are the corresponding non-hydrogen atom count (N_{NHA}), the rotatable bond count (N_R), and the effective rotor count (N_{ER}) found for the data series in **Figure 12**, panels **(a-c)**.
